# Single-cell profiling reveals epithelial and immune responses in BK polyomavirus–infected human kidney biopsies

**DOI:** 10.1172/jci.insight.198227

**Published:** 2026-03-12

**Authors:** Tess Marvin, Rachel Sealfon, Phillip J. McCown, Fadhl AlAkwaa, Evan A. Farkash, Edgar A. Otto, Felix Eichinger, Paul G. Cantalupo, Ping An, Rajasree Menon, Celine C. Berthier, Tavis J. Reed, Paula Arrowsmith, Lalita Subramanian, Kelly J. Shaffer, Silas P. Norman, Ramnika Gumber, Michael J. Imperiale, James M. Pipas, Olga G. Troyanskaya, Matthias Kretzler, Chandra L. Theesfeld, Abhijit S. Naik

**Affiliations:** 1 Lewis-Sigler Institute for Integrative Genomics and; 2 Princeton Precision Health Initiative, Princeton University, Princeton, New Jersey, USA.; 3 Center for Computational Biology, Flatiron Institute, New York, New York, USA.; 4 Department of Internal Medicine, Division of Nephrology, and; 5 Department of Pathology, University of Michigan, Ann Arbor, Michigan, USA.; 6 Department of Biological Sciences, University of Pittsburgh, Pittsburgh, Pennsylvania, USA.; 7 Department of Computational Medicine and Bioinformatics and; 8 Department of Microbiology and Immunology and Rogel Cancer Center, University of Michigan, Ann Arbor, Michigan, USA.

**Keywords:** Immunology, Nephrology, Virology, Organ transplantation, Transcriptomics

## Abstract

**INTRODUCTION:**

BK polyomavirus (BKV) infection is associated with injury and subsequent graft loss due to the extent of injury or rejection. However, the molecular mechanisms driving injury and subsequent adverse outcomes remain poorly understood.

**METHODS:**

In a cross-sectional study, single-cell RNA-seq from kidney allograft biopsies was used to assess cell type–specific responses between uninfected controls and 2 distinct phases of BKV infection: peaking (increasing viral blood titers) and resolving (decreasing viral titers following immunosuppression reduction).

**RESULTS:**

Genes upregulated in BK viral nephropathy (BKVN) were enriched for polyomavirus infection hallmarks, including ribosome biogenesis, translation, and energy restructuring. Additionally, enriched pathways included wound healing, cellular stress, antigen presentation and immune signaling. Even without BKVN (peaking BK viremia alone), epithelial cells expressed signatures for wound healing, cellular stress, and extracellular matrix remodeling. In vivo tubular cell responses at single-cell resolution were validated against single cell transcriptomic data of BKV-infected cells in a cell culture model. Despite similarities, in vivo tubular cells underwent metabolic adaptation favoring fatty acid oxidation and proinflammatory responses not observed in culture models, likely due to an absent innate and adaptive immune system. Despite lymphopenia and immunosuppressive therapies, the proportion of recipient-derived intrarenal adaptive immune cells was increased in biopsies associated with peaking viremia alongside activation of innate immune responses. Adaptive immune cells exhibited persistent inflammatory signaling and remodeling of energy metabolism during the resolving phase of infection.

**CONCLUSION:**

These not previously reported insights into BKV-associated injury may have implications for clinical management and improved allograft outcomes.

## Abstract 2

BK polyomavirus (BKV) is a common virus that can cause serious complications in kidney transplant recipients, leading to kidney damage and even transplant rejection. This study examines how BKV affects kidney cells and the immune system by analyzing kidney biopsy samples from transplant patients at different stages of infection

## Graphical abstract

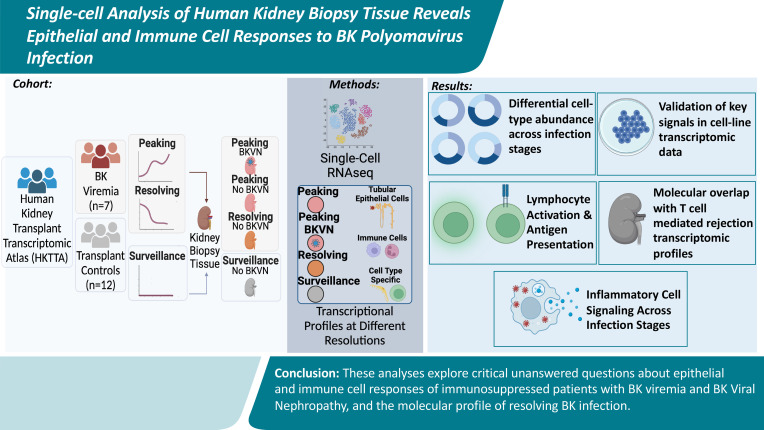

## Introduction

BK polyomavirus (BKV) is a common opportunistic pathogen that, following primary infection, establishes a persistent subclinical infection in most healthy adults (1). Often reactivating after kidney transplantation, BKV can cause extensive damage or loss of the allograft (2). The estimated prevalence of BK viremia in transplant recipients ranges from 17% to 31%, depending on local immunosuppression protocols and infection definitions (3, 4). The current standard of care to reduce the risk of developing BK viremia and nephropathy involves prospective monitoring and reduction of immunosuppression before significant allograft injury occurs (5, 6). However, the reduction of immunosuppression can lead to an increase in circulating donor-specific anti-HLA antibodies and rejection risk, associated with high rates of graft loss (7–10). We hypothesized that investigating molecular signals in epithelial and immune cells during the peaking and resolving phases of BK viremia, following immunosuppression reduction, will provide mechanistic insights into BKV mediated graft injury and the host immune response, both crucial for improving patient outcomes.

Primary BKV infection usually occurs during childhood, with > 80% seroprevalence in the adult population (8). In healthy people, BKV typically persists in the urinary tract (8). However, BKV and the closely related JC virus have been reported in circulating immune cells (11, 12). When BKV reactivates in a kidney transplant recipient, viral replication leads to enlarged nuclei due to G2/M arrest in infected cells, and intranuclear viral inclusions are observed (13). The lytic viral life cycle results in the death of tubular-epithelial cells, prompting viruria (viral shedding in the urine) and subsequent viremia (virus in the blood) (2). BK viral nephropathy (BKVN) causes significant morbidity due to extensive inflammation and fibrosis (14).

The viral life cycle and cellular response have been profiled in primary human kidney proximal tubular epithelial cells (15–19). These studies have been limited by their ex vivo nature, where the cytolytic destruction of infected cells hindered attempts to model persistent infection. Recently, resistant cell lines have been developed (20), enabling persistent BKV infection under specific conditions (21, 22). Infection at the single-cell level has been described in these primary cells in culture (23) but not in the context of the intact kidney, with dynamic immune system dynamics across infection phases.

This work investigated the host response to BK viremia and BKVN using transplant kidney biopsy tissue. Cell type–specific patterns of the host response to viral infection and intrarenal immune activation signatures across phases of infection were profiled using single-cell RNA-seq (scRNA-seq). These analyses enabled the exploration of many critical unanswered questions regarding epithelial and immune cell responses in kidney allografts of immunosuppressed patients with BKVN and during the peaking and resolving phases of BK viremia.

## Results

**Figure 1:**
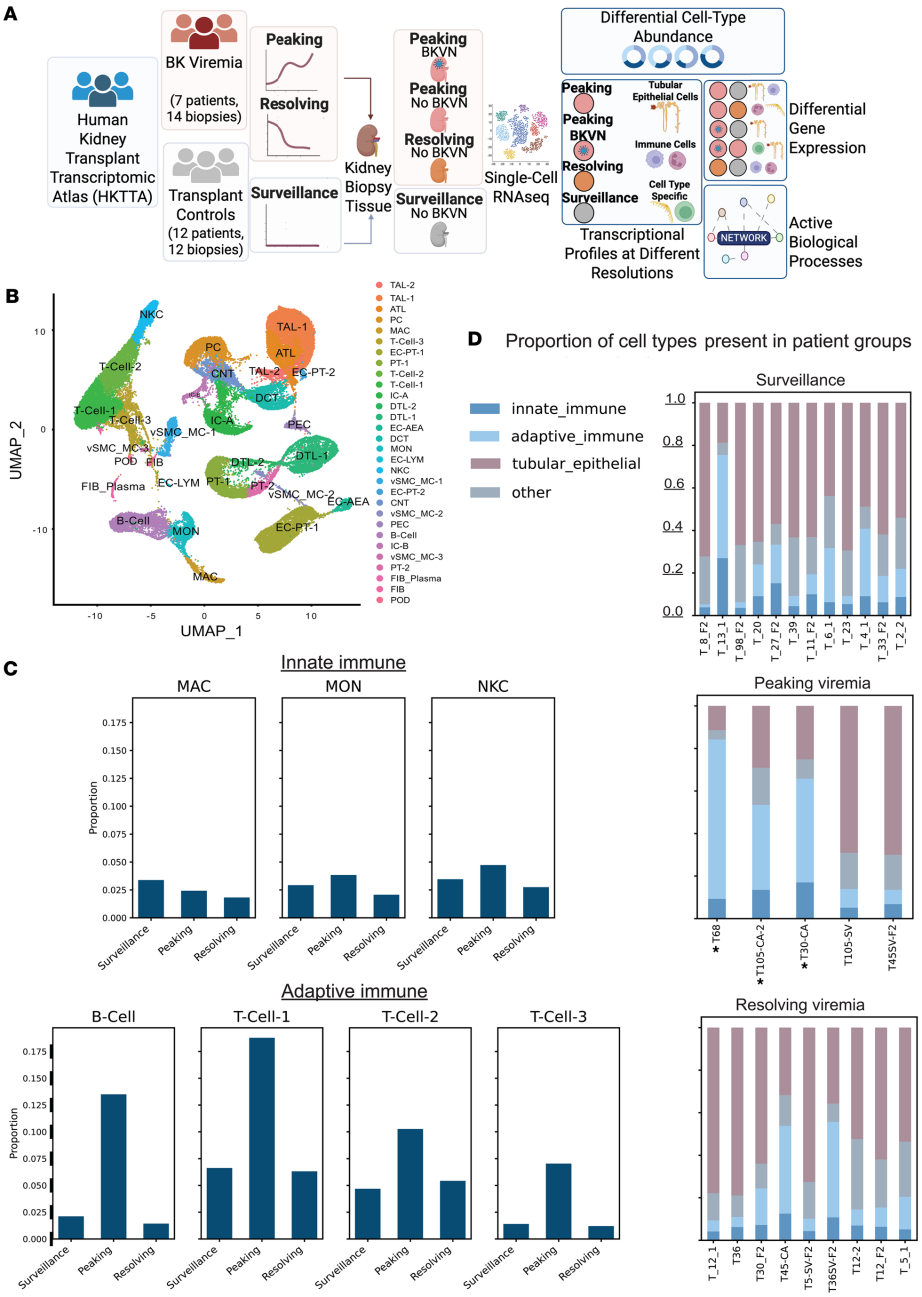
Single-cell RNA-seq of BKV and BKVN in transplant kidney biopsies. (**A**) Overview of analysis of BKV and BKVN in transplant kidney biopsies: 26 biopsies were selected to study the effects of BKV infection on allografts. Twelve surveillance biopsies, 5 from patients with peaking viremia in the blood, and 9 from patients with resolving viremia were subject to single-cell RNA-seq analysis. The transcriptomic analysis of the response to BKV and BKVN was determined for tubular epithelial and immune compartments. Differential cell-type abundances across patients and groups were assessed to indicate host-immune response to infection. Results were compared with the transcriptomic signals in the cell line BK infection model to determine which aspects of the in vivo signals are modeled in vitro and which components are unique to the response in the kidney. (**B**) UMAP of >63,000 sequenced single cells from surveillance, peaking viremia, and resolving viremia biopsies that passed quality thresholds. (**C**) The proportion of adaptive immune cells (B cell, T cell 1, T cell 2, T cell 3) contributing to peaking biopsies is greater in adaptive immune cell contribution to biopsies from surveillance and resolving patients even as peaking patients are treated with triple-immunosuppressive therapy with tacrolimus, mycophenolate mofetil, and prednisone. The proportion of innate immune cells (MAC, MON, NKC) contributing to peaking biopsies is not significantly enriched compared with other infection stages. (**D**) Proportion of cell-type class present in biopsies (asterisks indicate biopsies from patients with BKVN, a subset of peaking viremia patients). ATL, ascending thin limb; CNT, connecting tubule; DCT, distal convoluted tubule; DTL-1, descending thin limb 1; EC-AEA, endothelial cell afferent/efferent arteriole; EC-LYM, endothelial cell lymphatic; EC-PT, endothelial cell peritubular capillary; FIB, fibroblast; FIB_Plasma, fibroblast/plasma cell; IC-A, intercalating cell A; IC-B, intercalated cell B; MAC, macrophage; MON, monocyte; NKC, natural killer cell; PC, principal cell; PEC, parietal epithelial cell; POD, podocyte; PT-1, proximal tubule 1; PT-2, proximal tubule 2; TAL-1, thick ascending limb; vSMC_MC, vascular smooth muscle cell/muscle cell.

The analytical approach for this study is summarized in Figure 1A.

### Participant clinical characteristics

Participants clinical characteristics at the time of biopsy are presented in Table 1. Biopsy samples from 7 kidney transplant recipients in the Human Kidney Transplant Transcriptomic Atlas (HKTTA) were collected at different phases of BKV infection. Abbreviations used for each grouping are provided in Table 2 with additional details in the Methods section. Viral loads in allPeak (see methods section for definitions) ranged from 3,524 to 44,600 viral genome copies/mL, while viral loads in resBKVir ranged from 500 and 4,100 copies/mL. High viral loads and nonzero PVL scores are associated with BKVN (24). Both patients in the peakBKVir group had BK viral loads below 10,000 copies/μL and PVL scores of zero, consistent with their histologic diagnosis of no BKVN (25). In contrast, 2 of the 3 patients in the BKVN group had high viral loads exceeding 10,000 copies/μL and nonzero PVL scores, consistent with BKVN. These 3 biopsies all show SV40 staining, confirming BKVN. Table 3 presents the Banff 2022 scores for all patients with BK viremia, while the Banff scores for surveillance biopsy patients have been published previously (26). Table 4 summarizes comparisons across the groups referenced in the analysis below.

### Characterization of cell types in BKV infection phases

After quality control, 63,167 cells from 26 kidney biopsies were retained for analysis. Among these, 46,211 cells were derived from 14 BKV-infected samples; including 19,080 cells from BKVN; 4,467 from peakBKVir; and 22,664 from resBKVir. Control biopsies (surveillance [SURV]) contributed 16,956 cells. Unsupervised clustering identified 30 distinct cell populations (Figure 1B), representing major kidney cell types. All patients contributed cells from both the medulla and cortex, capturing a broad spectrum of renal cell types. Each cluster included cells from all phases of BK infection and controls (Supplemental Figure 1; supplemental material available online with this article; https://doi.org/10.1172/jci.insight.198227DS1).

The proportion of adaptive immune cells was significantly higher in BKVN (*n* = 3 biopsies) and peakBKVir (*n* = 2 biopsies) compared with SURV (*n* = 12 biopsies) and resBKVir (*n* = 9 biopsies). In contrast, the proportion of innate immune cells remained stable across all patient groups (Figure 1C). Notably, the adaptive immune cell compartment was expanded in BKVN (Figure 1D) relative to peakBKVir. Immune cells were predominantly recipient-derived, with only 1% carrying donor sex-specific markers (RPS4Y1 in male donors, XIST in female donors) in patients where the donor and recipient were of different sexes.

### Epithelial cell response to peaking BK viremia and BKV nephropathy

**Figure 2:**
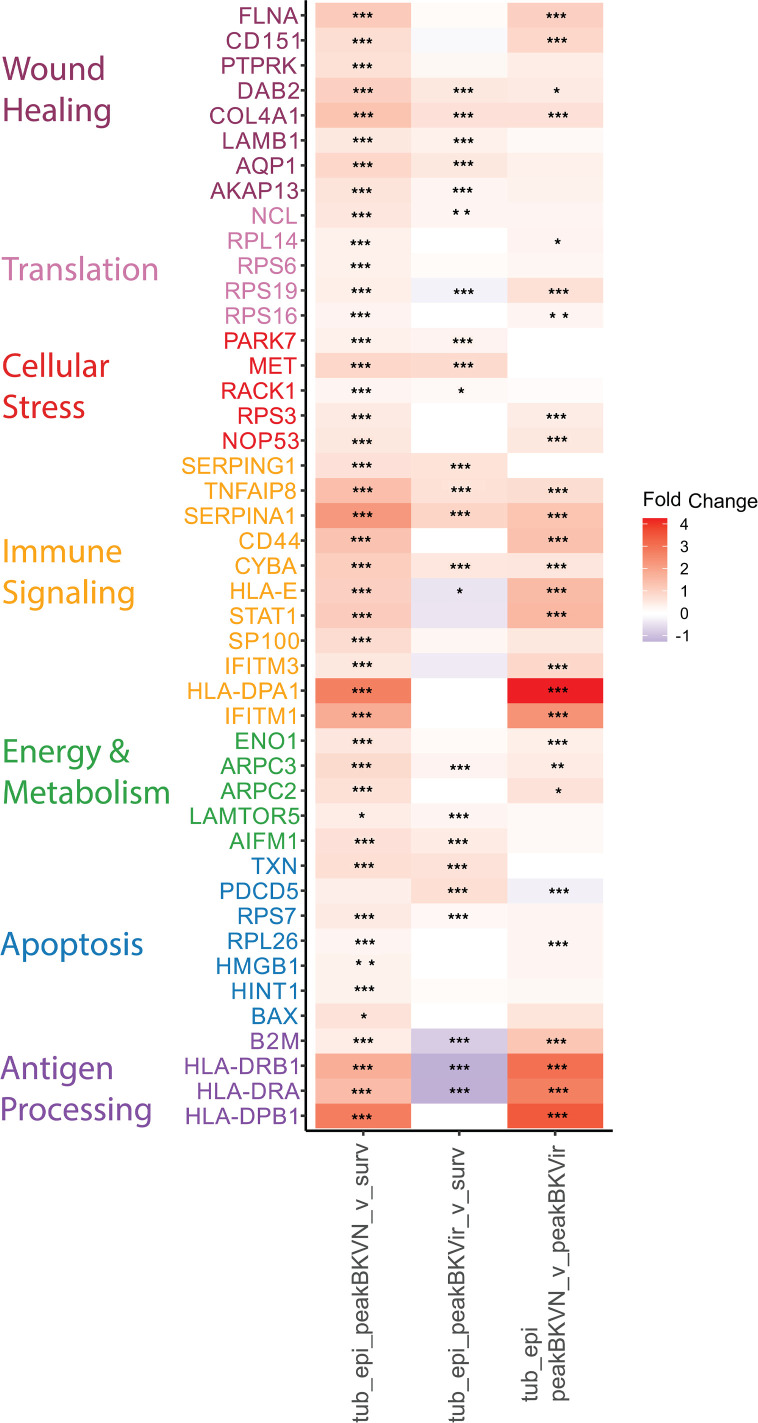
Expression signature of tubular epithelial cells with BKVN and BKVir compared with surveillance biopsies. The transcriptional response of tubular epithelial cells to peakBKVir and BKVN was assessed by comparing gene expression profiles across 3 conditions: peakBKVir versus SURV, BKVN versus SURV, and BKVN versus peakBKVir. PeakBKVir cells exhibited transcriptional signatures indicative of cellular stress, apoptosis, and energy metabolism remodeling, with increased expression of genes associated with oxidative stress responses, metabolic adaptation, and stress signaling pathways. In contrast, BKVN demonstrated a distinct immune activation profile, including the upregulation of antigen processing and presentation genes (*HLA-DPA1*, *HLA-DPB1*, *HLA-DRB1*, *B2M*) and IFN-stimulated genes (*IFITM1*, *IFITM3*, *STAT1*), along with pathways related to inflammation, wound healing, and extracellular matrix remodeling. The comparison of BKVN and peakBKVir revealed nephropathy-specific responses beyond those seen in peakBKVir, including heightened immune signaling and epithelial injury responses. Statistical significance is denoted as follows: ***P < 0.001, **P < 0.01, and *P < 0.05.

The tubular epithelial cells of the kidney are considered the site of primary BK infection and prolonged persistence. Molecular signals upregulated in tubular epithelial cells with BK viremia without nephropathy were determined by comparing peakBKVir to SURV biopsies. To evaluate the biological signals in the differentially expressed genes, we used a network-based gene set enrichment analysis that enables pathway-level assessments by expanding the DEG gene lists (that may have sparse representation of pathways due to statistical noise) through data-driven definition of functionally related genes (27–29) (Supplemental Figure 2, Methods). PeakBKVir cells exhibited significant enrichment and upregulation of stress signaling, wound healing, and extracellular matrix remodeling. Furthermore, there was increased enrichment of apoptosis and energy metabolism pathways (Figure 2, Supplemental Figure 3, Table 4, Supplemental Table 2A [DEGs], and Supplemental Table 2B [enriched processes]), with notable increases in genes involved in oxidative stress responses and metabolic adaptation. To understand the effect of direct infection on epithelial cell signaling cascades, the transcriptomic profiles of tubular epithelial cells from BKVN biopsies and SURV biopsies were compared (Figure 2 and Supplemental Table 3, A and B). Genes upregulated in BKVN were enriched for polyomavirus infection hallmarks, including ribosome biogenesis, translation, and energy restructuring (Figure 2 and Supplemental Figure 3). Additionally, the BKVN signature displayed strong activation of immune-related pathways, particularly antigen processing and presentation, with increased expression of multiple HLA class I and II molecules and IFN-stimulated genes but no increase in the expression of IFN-γ transcript (Figure 2) suggesting response to an extracellular source of IFN-γ. Wound healing processes were also upregulated, as indicated by increased expression of extracellular matrix components and cell migration regulators. Immune-related signaling pathways, including those associated with inflammation, were elevated, alongside key mediators of apoptotic regulation and protein homeostasis (Figure 2 and Supplemental Figure 3).

### Comparison of transcriptional signatures of epithelial cells in patients with BKV nephropathy versus those with peaking BK viremia

The mechanisms specifically upregulated in nephropathy were elucidated by comparing BKVN with peakBKVir (Supplemental Table 4A [DEGs], Supplemental Table 4B [enriched processes], and Supplemental Figure 4). Notably, BKVN exhibited a stronger innate immune response, characterized by significant upregulation of antigen processing and presentation genes (*HLA-DPA1*, *HLA-DPB1*, *HLA-DRB1*, *B2M*) and IFN-stimulated genes (*IFITM1*, *IFITM3*, *STAT1*). In contrast, several HLA genes and antigen processing genes were downregulated in patients in the peakBKVir group. The BKVN signature was also distinguished by the upregulation of several immune-related signaling pathways, including SMAD proteins, Rho proteins, protein kinase A, receptor protein tyrosine kinase, and Ras protein signal transduction (Figure 2 and Supplemental Figure 3). Additionally, pathways related to wound healing and extracellular matrix remodeling were more significantly enriched in BKVN, suggesting a more extensive epithelial response to injury and repair processes (Figure 2 and Supplemental Figure 4). This distinct transcriptional profile underscores the role of immune activation and antigen presentation as defining features of BKVN beyond peakBKVir.

### Comparison of transcriptional signatures of tubular epithelial cells of patients with BKV nephropathy with infected cells in culture

Cultured renal proximal tubular epithelial (RPTE) cells are an important model for studying BKV infection, and we investigated if the signals defined in this model were observed in vivo. A hallmark of BKV-infected RPTE cells is an increase in the proportion of cycling cells (23). There was a significantly higher proportion of cells assigned to the G2/M or S phase (*P* = 0.0008) in allPeak compared with SURV samples (Supplemental Figure 5). This finding suggests BK polyomavirus infection induces widespread cell cycle dysregulation in vivo in human proximal tubules.

**Figure 3:**
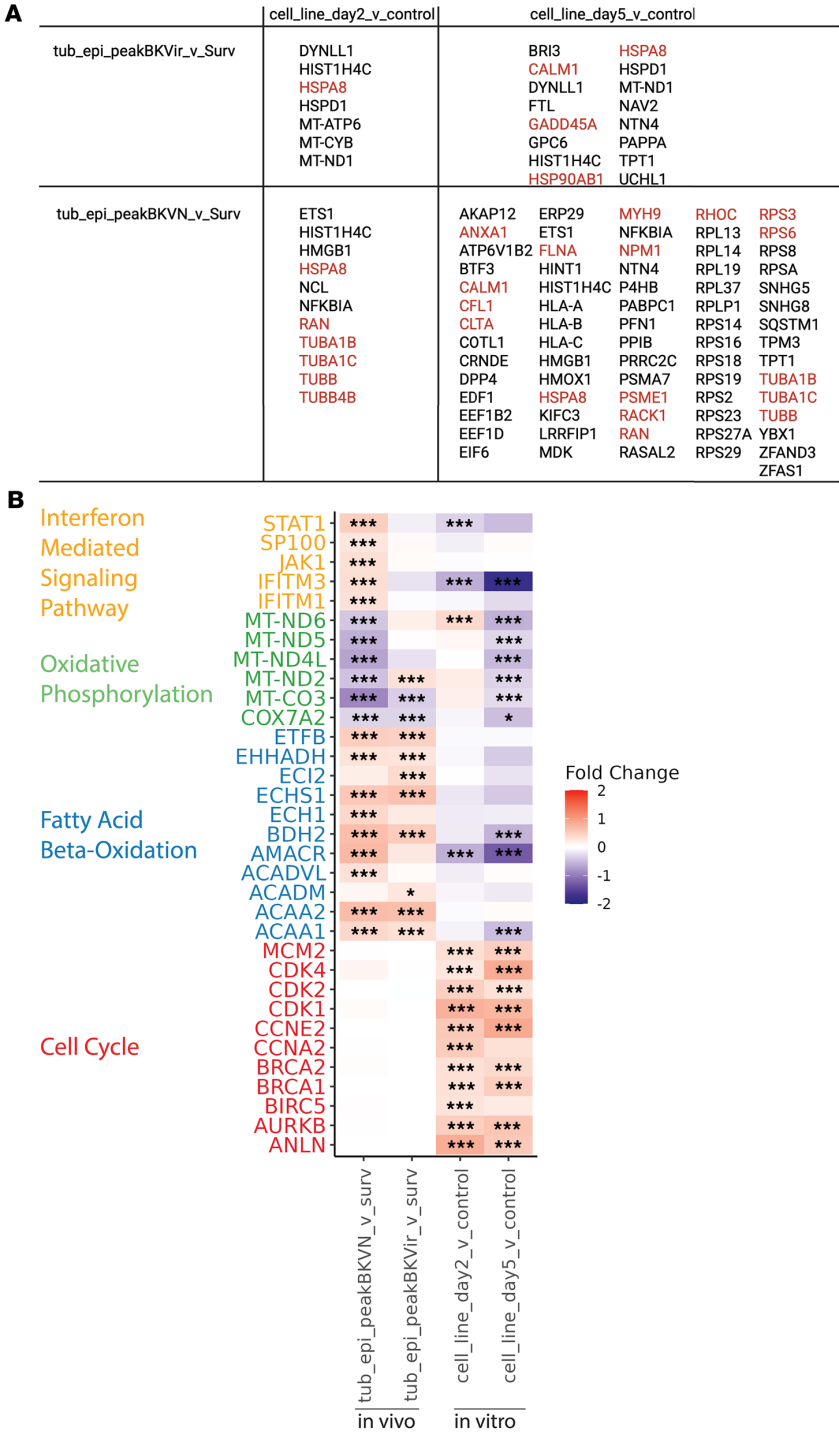
Similarities and differences between in vivo transcriptional signatures and in vitro transcriptional signatures in cell lines. (**A**) Genes that are significantly upregulated across both cell lines and in vivo transcriptional signatures. Markers that are annotated to the “cell cycle” GO term are highlighted in red. (**B**) Genes involved in processes differing across in vivo and in vitro signatures. Many additional cell cycle genes are upregulated in the infected cell line but not differentially regulated in tubular epithelial cells in vivo. Fatty acid β-oxidation markers are robustly upregulated in vivo but not in the cell line. Some oxidative phosphorylation markers are significantly downregulated in tubular epithelial cells from patients with peaking BKVN but not in other comparisons. A group of IFN-mediated signaling pathway genes are significantly upregulated in tubular epithelial cells from patients with peaking BKVN but not in other comparisons. The statistical significance of each fold change is denoted by *P_adj_ < 0.05, **P_adj_ < 0.01, ***P_adj_ < 0.001.

We next evaluated if gene expression changes observed in infected cell lines profiled by An et al. were also present in the transcriptional signatures identified in single-cell data from epithelial cells in vivo (23) (Figure 3 and Supplemental Tables 5 and 6). A significant overlap was observed in BKV-associated transcriptional changes between BKV-infected RPTE cells in culture and tubular epithelial cells from peaking BKVN human biopsy samples (Figure 3A). This concordance confirms that numerous stress response and cytoskeletal genes are significantly upregulated in both infected cell lines and in vivo tubular epithelial cells, thereby validating our single-cell findings and reinforcing the role of these pathways in the epithelial response to infection (Figure 3A).

Genes involved in cellular stress responses, including protein homeostasis (*HSPA8*, *HSPD1*, *HSP90AB1*, *PSME1*), oxidative stress regulation (*HMOX1*), inflammation and signaling (*NFKBIA*, *HMGB1*), and DNA damage repair and cell cycle regulation (*GADD45A*), were consistently upregulated across both comparisons, underscoring their role in cellular adaptation to infection. Additionally, the IFN-γ transcription was absent in epithelial cells in both the in vivo and in vitro settings, suggesting a conserved epithelial response to BKV infection (17, 23). However, genes responsive to IFN signaling are significantly upregulated in tubular epithelial cells from patients with peaking BKVN but not in the infected RPTE cells (Figure 3B).

Beyond these shared stress response pathways, we observed differences between in vivo and in vitro transcriptional signatures, particularly in cell cycle regulation, metabolic adaptation, and immune responses (Figure 3B). Many cell cycle regulators (*CDK4*, *CDK2*, *CDK1*) are strongly upregulated in infected RPTE cells but not differentially expressed in tubular epithelial cells in vivo. This suggests that while BKV infection induces proliferative signaling in both settings, in vitro conditions may amplify cell cycle activation without regulatory constraints present in the tissue. In contrast, fatty acid β-oxidation genes are robustly upregulated in vivo but show no significant regulation in the infected cell line, suggesting that metabolic shifts in response to BKV infection are unique to the tissue microenvironment (Figure 3B).

### Immune responses to BKV

While immune activation is necessary to clear active BKV infection, it also poses the risk of immunopathologic damage to the kidney. Thus, it is crucial to understand both immune pathways that are important in response to viruses and pathways that may result in collateral allograft damage. All patients were under maximal immunosuppression regimen and demonstrated leucopenia (white blood cell count for all patients below 4,000 cells/μL; Table 1). However, peakBKVir and BKVN biopsies exhibited intrarenal increases in immune cells and activation signatures, indicating an intrarenal cellular immune response (Figure 1C).

#### Comparison of peaking BK viremia and transplant SURV biopsies.

**Figure 4:**
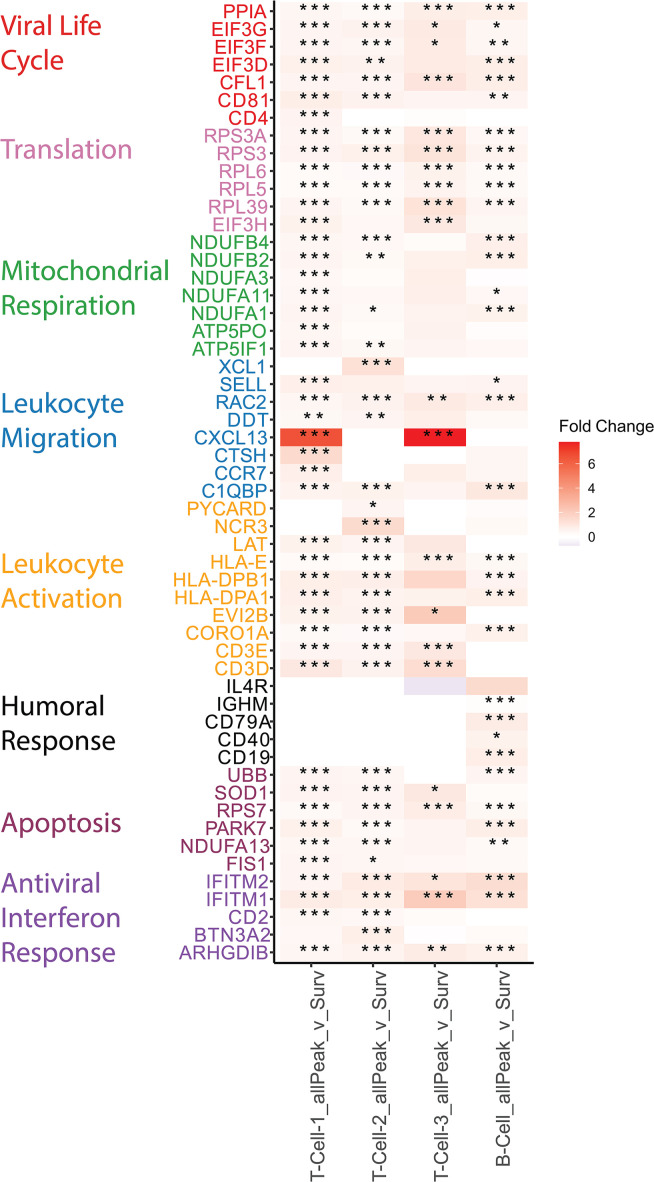
Expression signatures from T and B cells, comparing allPeak to surveillance biopsies. The transcriptional response of adaptive immune cells during BK viral infection was assessed by comparing allPeak gene expression profiles to surveillance. T cells exhibited upregulation of genes involved in protein translation, activation, and migration, suggesting a proliferative T cell response. Additionally, effector functions, antiviral IFN signaling, apoptotic pathways, and mitochondrial respiration were enriched. In B cells, pathways related to antigen processing and presentation, leukocyte activation, and proliferation were enriched, alongside increased expression of genes involved in protein translation and ribosome biogenesis, suggesting active cell growth. Additionally, B cells exhibited significant upregulation of genes associated with the humoral immune response, including signaling through *CD79A* and *CD40*. The statistical significance of each fold change is denoted by *P_adj_ < 0.05, **P_adj_ < 0.01, ***P_adj_ < 0.001.

T cells from allPeak biopsies exhibited enrichment of genes upregulated in protein translation, activation, and migration suggestive of a proliferative T cell response; furthermore, effector functions were upregulated in addition to antiviral IFN signaling, apoptotic signaling, and mitochondrial respiration (Figure 4, Supplemental Figure 6, Supplemental Tables 7A–9A, and Supplemental Tables 7B–9B). This signal in allPeak biopsies was driven by the BKVN biopsies; we observed identical signals in T cells when comparing BKVN to SURV. This is likely due to the associated intrarenal immune compartment expansion (Figure 1, C and D). Expression patterns suggest T Cell-1 cells are Th17 cells, key regulators of inflammatory responses and tissue remodeling (Supplemental Figure 7) (30). T Cell-2 cells represent CD8^+^ cytotoxic T cells responsible for potent effector functions (Supplemental Figure 7) (31). T Cell-3 cells exhibit a Treg phenotype (Supplemental Figure 7) (32). T Cell-1 cells (Th17 cells) likely contribute to inflammatory responses within the graft, promoting tissue remodeling and immune activation (Supplemental Figure 6A). T Cell-2 cells (CD8^+^ T cells) are engaged in the direct killing of target cells, as evidenced by the upregulation of pathways related to T cell–mediated cytotoxicity (Supplemental Figure 6B). The T Cell-3 cluster, identified as Tregs, showed enrichments linked to immune suppression and homeostasis. Notably, protein ubiquitination pathways suggest posttranslational regulation of key signaling molecules, essential for FOXP3 stability, which drives Treg-mediated immunosuppression (Supplemental Figure 6C) (33).

#### Comparison of peaking BK viremia versus transplant control biopsies.

Like T cells, B cells were more numerous in allPeak than SURV, suggesting a coordinated B and T cell response. Functional enrichment analysis indicated that pathways induced in B cells from allPeak compared with SURV included antigen processing and presentation (major histocompatibility complexes) and several processes associated with leukocyte activation and proliferation: actin cytoskeleton organization, chemotaxis, and leukocyte cell-cell adhesion (Figure 4, Supplemental Figure 6D, and Supplemental Table 10, A and B). Many induced genes were relevant to the biosynthetic capacity of B cells—protein translation and ribosome biogenesis—indicating that cell growth was activated and congruent with the observed expansion of the B cell population in allPeak. Furthermore, when comparing B cells from the allPeak to SURV, a significant enrichment of genes associated with the mitochondrial organization and the electron transport chain was observed. In addition, several signaling pathways were stimulated, including the p38 mitogen-activated protein kinase (p38MAPK), I-κB kinase/NF-κB, phosphatidylinositol 3-kinase, Rho protein, and c-Jun N-terminal kinase (JNK) cascades (Supplemental Figure 6D). Direct comparison of B cells from resBKVir to SURV revealed signatures of ongoing defense against virus and infection (Supplemental Figure 8 and Supplemental Table 11, A and B). Thus, despite entering the resolving phase, B cells continue to exhibit significant upregulation of genes associated with ribosome biogenesis, leukocyte activation, and apoptosis. Key ribosomal genes remain differentially expressed, indicating ongoing protein synthesis to support sustained humoral response (Supplemental Figure 8). Genes involved in leukocyte activation also show persistent upregulation and differentially expressed apoptosis-related signatures.

#### Comparison of peaking BK viremia versus control biopsies.

**Figure 5:**
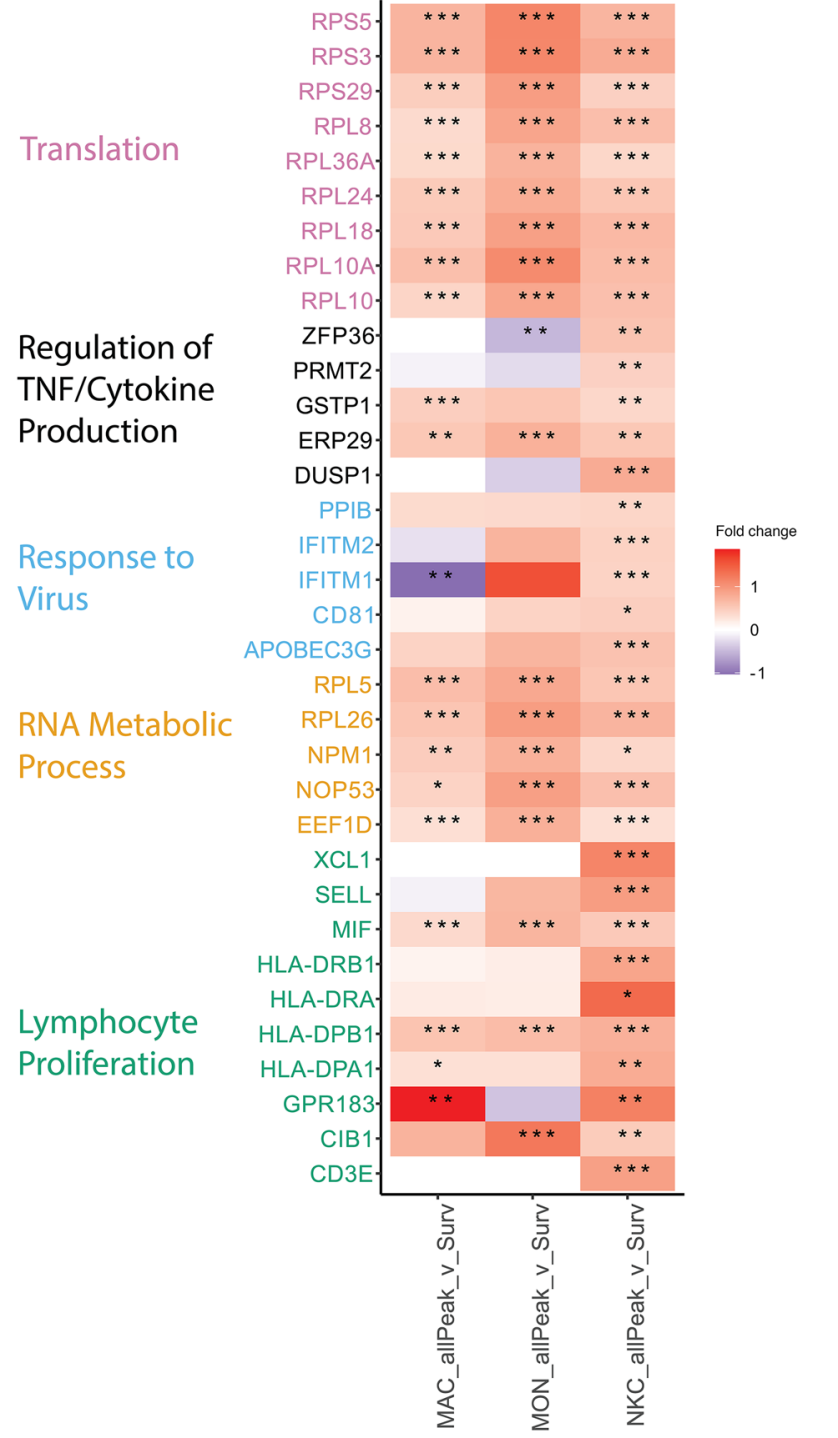
Innate immune cells transcriptional responses with peaking BK viremia compared with surveillance biopsies. The transcriptional landscape of innate immune cells from allPeak biopsies was assessed by comparing gene expression profiles to SURV. Despite no expansion of the innate immune cell population in allPeak, genes associated with translation, RNA metabolic processes, and lymphocyte proliferation were upregulated across innate immune cell types, suggesting heightened biosynthetic and proliferative activity. Beyond these shared activation signatures, distinct pathways were enriched in specific innate immune cell types. NKCs from allPeak exhibited the most pronounced upregulation of genes related to antigen processing and presentation, regulation of cytokine and TNF production, and response to viral infection, indicating a strong antiviral and immunoregulatory state. The statistical significance of each fold change is denoted by *P_adj_ < 0.05, **P_adj_ < 0.01, ***P_adj_ < 0.001.

We did not observe an expansion in cell numbers of the innate immune cell population in allPeak biopsies (Figure 1, C and D); however, genes associated with translation, RNA metabolic processes, and lymphocyte proliferation were upregulated across innate immune cell types in allPeak compared with SURV (Figure 5, Supplemental Tables 12A–14A, Supplemental Tables 12B–14B, and Supplemental Figure 9).

In addition to the shared activation signatures, several pathways were uniquely induced in specific innate immune cell types. NK cells (NKC) from allPeak demonstrated the most robust expression of genes related to antigen processing and presentation, regulation of cytokine and TNF production, and response to viral infection (Figure 5 and Supplemental Figure 9C). Biopsies in patients with BKVN represent a more translationally active state than patients with BK viremia alone in innate immune cell types, with widespread upregulation of ribosomal genes (Supplemental Figure 10). Genes involved in immunological synapse formation were also upregulated in BKVN monocytes compared with peakBKVir (Supplemental Figure 10). Notably, *CXCL10*, a chemokine expressed by macrophages and monocytes in the kidney (GTEX) and known to be elevated in the urine of patients with BKV, was downregulated in intrarenal monocytes and macrophages in allPeak biopsies (34, 35).

#### Comparison of intrarenal T cell signature of patients with peaking BK viremia and BKVN with T cell mediated rejection signatures in 2 studies.

**Figure 6:**
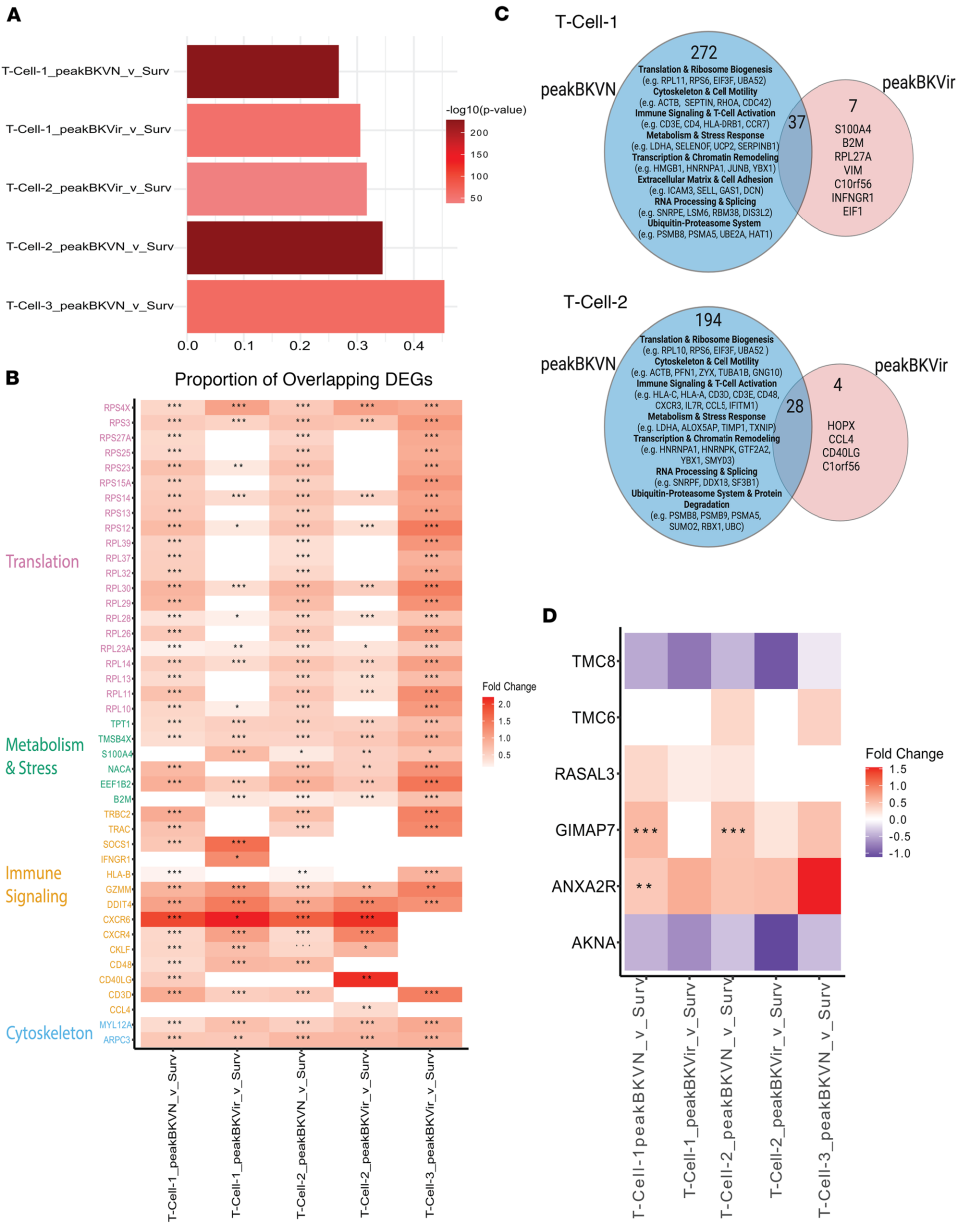
Expression of TCMR-associated genes in T cell populations of patients with peaking BKV. (**A**) Enrichment of DEGs from all T cells and their subtypes in patients with BKVN or peakBKVir against a validated TCMR gene signature derived from bulk RNA-seq of biopsies with TCMR compared with non-TCMR samples that include normal, injury, and antibody mediated rejection biopsies (36). The signature included all differentially expressed genes, up- and downregulated. Enrichment was assessed using Fisher’s exact test, revealing significant overlap across all T cell subsets. T Cell-3 peakBKVir was excluded due to insufficient cells. (**B**) Heatmap showing expression programs induced across all T cell subsets overlapping with TCMR DEGs, highlighting pathways related to translation, ribosome biogenesis, metabolism, stress, immune signaling, and cytoskeletal regulation. The enrichment of these pathways was more robust in BKVN than in peakBKVir. (**C**) To investigate the transcriptional differences between peak BKVN and peak BKVir within T cell subsets, we performed an overlap analysis focusing on DEGs that are shared with the TCMR signature. Functional annotations highlight key biological pathways associated with BKVN genes that overlap the TCMR signature. (**D**) Heatmap depicting the expression of 6 TCMR-associated genes (*AKNA*, *ANXA2R*, *GIMAP7*, *RASAL3*, *TMC6*, and *TMC8*) in T cell populations from BK polyomavirus (BKV) nephropathy patient samples. *ANXA2R* and *GIMAP7* were significantly upregulated, while *AKNA* and *TMC8* were downregulated. The differential expression of these genes may indicate potential overlaps and differences between viral immune responses and alloimmune rejection mechanisms in kidney transplantation. Statistical significance of each fold change is denoted by *P_adj_ < 0.05, **P_adj_ < 0.01, ***P_adj_ < 0.001. Underlying data was obtained from the paper by Zhang et al. (37).

To investigate the molecular overlap between TCMR-associated gene signatures and intrarenal T cell responses in BK biopsies, we compared signatures between published studies and T cells from peaking samples. We first compared a TCMR signature from a study that looked at differentially expressed genes between TCMR and non-TCMR controls that included samples with inflammation, injury, and other rejection characteristics derived from Cortes Garcia et al. (36). We then performed an enrichment analysis comparing DEGs of all T cells and their subtypes from patients with BKVN and peakBKVir. Genes from both datasets were filtered to include only those with significant differential expression, and overlap was determined based on the concordant directionality of expression. Enrichment of TCMR-associated genes within the BKV transcriptomic signature was assessed using Fisher’s exact test, revealing significant enrichment across multiple T cell subsets (Figure 6A). T Cell-3 peakBKVir was not included in this analysis due to insufficient cells. The overlapping signature included expression programs across T cell subsets, including pathways related to translation ribosome biogenesis, metabolism, stress, lipid transport, immune signaling, extracellular matrix organization, and cytoskeletal regulation (Figure 6B). The enrichment of these expression programs was greater in T cells from patients with BKVN than in patients with peakBKVir, implying that BKVN induces programs more similar to TCMR than peakBKVir. When we examined the TCMR signature genes that overlapped BK DEGs, we observed that some were unique to peakBKVir, but a higher proportion were unique to BKVN; these genes were involved in antigen processing and presentation, T cell proliferation, cytoplasmic translation, and protein stability (Figure 6, B and C).

We compared the T cell signals in peaking biopsies to a second study that characterized TCMR rejection signals specifically from biopsies that showed expanded diverse populations of T cell CDR3 clonotypes associated with rejection (37). The report defined a 6-gene set (*AKNA*, *ANXA2R*, *GIMAP7*, *RASAL3*, *TMC6*, and *TMC8*) that showed increased expression across multiple cell types in rejection biopsies. In our analysis, none of these genes were upregulated in T cells from peakBKVir samples, and only 2 showed significant upregulation in BKVN (*ANXA2R* and *GIMPA7*). *ANXA2R* was upregulated in the T Cell-1 cluster of patients with BKVN, and *GIMPA7* was upregulated in the T Cell-1 and T Cell-2 clusters of patients with BKVN (Figure 6D).

### Detection of BKV in kidney cells using scRNA-seq

The analysis of single cells enabled us to profile cell types with BK viral transcripts. We found BK viral transcripts in a subset of cells via scRNA-seq, including both tubular epithelial and immune cell clusters (Supplemental Figure 11, A and B). The detected transcripts included early-expressed small and large T antigens and late-expressed VP1, VP2, and agnoprotein. Reads were sparse, with low mean transcript counts per cell, and were absent in control or resolving BK samples. This low detection rate is in line with prior reports for RNA-seq of diverse in vivo viral infections (38–40). BLAST and SNP analyses confirmed that all viral reads mapped to human polyomavirus subtype 1 and that each affected biopsy carried a unique BKV strain (Supplemental Figure 12).

IHC and transmission electron microscopy identified BK viral proteins and virions in tubular epithelial cells, consistent with previous studies (Supplemental Figure 11B and Supplemental Figure 13). While some viral reads were detected in cells from immune clusters, this observation requires further validation using orthogonal techniques. In vitro studies have demonstrated that lymphocytes can transmit BKV to renal tubular cells (41, 42). Furthermore, previous studies have identified BKV in circulating immune cells (11, 12).Given the limited number of detected transcripts and exhaustion of tissue sections in the studied patients, these findings are best interpreted as preliminary, and additional targeted approaches will be necessary to confirm whether circulating and infiltrating immune cells harbor viral RNA and allow for viral replication in vivo during infection.

## Discussion

This cross-sectional study leveraged a retrospective cohort of kidney transplant recipients, incorporating detailed clinical, histological, and molecular data from individuals with and without BK viremia. This approach enabled the investigation of intrarenal, cell type–specific molecular processes associated with different phases of BKV infection. The findings provide insights into tubular epithelial and immune cell responses in vivo during peaking BK viremia, both with (BKV nephropathy, BKVN) and without nephropathy (peakBKVir), as well as during the resolving phase of viremia (resBKVir). Additionally, they offer a comparative perspective on how these responses differ from in vitro models.

Even without BKVN, individuals with peakBKVir exhibited enrichment of transcriptional programs related to stress, wound healing, extracellular matrix remodeling, apoptosis, and energy metabolism. These data suggest that, even without detectable virus in the kidney, host tubular epithelial cells respond to the presence of viremia. In patients with BKVN, many of these transcriptional programs were amplified, along with upregulation of innate immune signaling, antigen presentation and processing, IFN-stimulated genes, and wound-healing pathways. Both patients in the peakBKVir group had BK viral loads below 10,000 copies/μL and a PVL score of 0 (25). In contrast, 2 of the 3 patients in the BKVN group had viral loads exceeding 10,000 copies/μL, consistent with previous data evaluating the relationship between viral load and the risk of developing BKVN (24).Taken together, our data suggest increased tubular injury and immune response transcriptional programs across the spectrum of infection from those with no BK infection, those with peakBKVir and those with established BKVN. Furthermore, the data also support that even low-grade BK viremia may not be entirely clinically benign and highlight the need for improved noninvasive biomarkers not prone to sampling error, as occurs with kidney biopsies, to definitively rule out BKVN.

There were important differences in BKV infections in the kidney compared with RPTE cells in culture. Similar to cell culture models, which lack IFN-γ transcription, there was no increased IFN-γ transcription in tubular cells. However, there was increased IFN-γ–mediated signaling in tubules of patients with BKVN, suggesting that the signal originates from an extracellular source. These data suggest that increased proapoptotic and proinflammatory signaling in kidney allografts may arise from immune-epithelial cell crosstalk, a phenomenon not captured in previous cell culture models. Another noteworthy difference was that in vivo tubular cells from patients with BKVN had increased expression of genes associated with fatty acid oxidation, suggestive of metabolic adaptation, a phenomenon not observed in cell culture models. We hypothesize that this metabolic shift in energy handling represents an adaptive response to sustain cellular energy homeostasis amid chronic viral infection, immune activation, and tissue stress.

T and B cells from peaking biopsies induced RNA regulatory processes, chemotaxis, energy metabolism, and cytokine production, suggesting an activated state. While B cells were similarly activated during the peaking and resolving phases of infection, those in the resBKVir group exhibited distinct inflammatory signaling and energy metabolism changes. Additionally, apoptotic pathway genes remained differentially expressed, suggesting persistent B cell turnover. These findings indicate that B cells in the resolving phase retain residual immune activity rather than fully returning to a quiescent state. Whether this B cell profile contributes to the development of donor-specific antibodies, as observed in many patients, remains an important question but is beyond the scope of this manuscript.

Despite their known role in BKV infection, we did not observe a notable expansion in cell numbers of the innate immune cell population in allPeak biopsies (Figure 1, C and D) when expressed as a proportion of all cells sequenced. However, genes signatures across innate immune cell populations demonstrated increased translation, RNA metabolic processes, and proliferative responses in allPeak compared with SURV (Figure 5, Supplemental Tables 12A–14A, Supplemental Tables 12B–14B, and Supplemental Figure 9) suggestive of an ongoing response to local BKV infection.

In addition to the shared activation signatures, several pathways were uniquely induced in specific innate immune cell types. NKC from allPeak demonstrated the most robust expression of genes related to antigen processing and presentation, regulation of cytokine and TNF production, and response to viral infection (Figure 5 and Supplemental Figure 9C). Biopsies in patients with BKVN represent a more translationally active state than patients with BK viremia alone in innate immune cell types, with widespread upregulation of ribosomal genes (Supplemental Figure 10). Genes involved in immunological synapse formation were also upregulated in BKVN monocytes compared with peakBKVir (Supplemental Figure 10).

While GTEx data indicate that monocytes and macrophages in the kidney are capable of producing CXCL10, scRNA-seq in BK-infected kidneys reveals downregulation of CXCL10 transcripts in these cell types (34, 35). This discrepancy suggests that elevated urinary CXCL10 protein levels in BKVN do not primarily stem from local renal production. Rather, akin to the marked upregulation of CXCL10 protein in bronchoalveolar lavage fluid during COVID-19 — which signifies a potent IFN-driven systemic immune response to viral infection (43, 44) — urinary CXCL10 in BKVN likely arises from heightened circulating levels spilling over into the urine.

In addition to the assessment of epithelial and immune cell responses we evaluated how transcriptional profiles of T cells in BKVN and peakBKVir patients compared with 2 TCMR signatures: a broad DEG signature from bulk RNA-seq and a single immune cell type–resolved signature from rejection biopsies with expanded CDR3 clonotypes (36, 37). The TCMR signature from Cortes Garcia et al. showed significant broad overlaps with BKVN T cell signatures as has been shown in previous studies using microarray and RNA-seq (45–47). In comparison, Adam et al. used gene expression of 800 targeted genes using NanoString technology in 110 archival kidney biopsy samples and identified good discrimination between BKVN and TCMR (48). In their study the Polyomavirus 5-gene set effectively differentiated BKVN from TCMR in the validation cohort, achieving high diagnostic accuracy (AUC = 0.992); however, the immune-related gene sets showed poor performance for differentiation (AUC ≤ 0.720), suggesting that discrimination between an overlapping immune response in TCMR and BKVN remains challenging.

In the higher resolution single cell type–derived rejection signature from Zhang et al., we observed that 2 of the 6 genes (*ANXA2R* and *GIMPA7*) were significantly upregulated in T cells from BKVN biopsies (37). Notably both these genes are relevant to T cell biology. ANXA2R expression was shown to correlate with high immune cell infiltration in the kidney in TCMR datasets and was a prognostic marker for kidney failure (37). While, *GIMAP7* is a GTPase involved in lymphocyte survival and has been implicated in T cell persistence (49, 50). Thus even at a single cell resolution our study was not able to discriminate TCMR signatures from T cell responses in BKVN. Thus, differentiating pure TCMR from BKVN T cell signatures, may require larger BKVN cohorts and multiomics integration to refine classifiers and mitigate issues like sample variability or undetected pathologies.

Despite the low number of viral reads detected in infected cells we observed a significant overlap in the transcriptional profile of epithelial cell responses between our dataset and the cell culture model by An et al., indicating that similar pathways are activated, regardless of viral read numbers (23). The low read counts may be due to sequencing depth limitations or in vivo regulatory constraints that influence viral transcript abundance. For example, in a previous study Abend et al. demonstrated the inhibitory effect of extracellular IFN-γ on BK viral replication in vitro (17). Our transcriptional data are consistent with activation of IFN-stimulated genes in tubular cells from an external source of IFN-γ. While we detected viral reads in immune cells, whether these reads represent replicative infection, a consequence of phagocytosis, or failed viral replication remains unclear and remains an open-ended question.

Despite the strengths of our study, key limitations include its cross-sectional retrospective design using University of Michigan data on BKV infection patients, with sequential biopsies before and after immunosuppression reduction largely unavailable (except 2 cases), prompting categorization into peaking or resolving viremia groups. Additionally, the absence of simultaneous biofluid collection during biopsies hindered correlations with circulating or urinary biomarkers.

This work highlights the value of multistage sampling and lays groundwork for future longitudinal scRNA-seq studies to map infection trajectories and rejection risks. Concurrent biofluid analyses could yield noninvasive biomarkers to differentiate BKVN from viremia alone and guide immunosuppression, enhancing outcomes.

## Methods

### Sex as a biological variable.

Both male and female participants were enrolled and included in the analysis.

### Tissue procurement and processing.

This retrospective study used kidney biopsy samples from patients enrolled in the HKTTA at the University of Michigan (IRB HUM00150968) collected at the time of their clinically indicated biopsies or during their 3-,6-, and 12-month posttransplant protocol biopsies (26). A single research core was extracted with a 16-gauge needle along with standard-of-care biopsy cores. One of 3 pieces of the research core (~1 mg) was transferred into CryoStor CS10 freeze media (BioLife Solutions) for scRNA-seq analysis.

Seven patients with BKV (14 biopsy samples) were identified from the HKTTA registry. Five samples were taken when BKV blood titers were rising (peaking BKV, allPeak), and 9 when the BKV blood titers were falling (resolving BK, resBKVir). Patients with peaking BKV were divided into those with nephropathy (BKVN) and those without (peakBKVir). Importantly, individual patients were sampled at different phases of infection, with 2 patients contributing biopsies to both the peaking and resolving groups. In contrast, others were only profiled within a single phase. All patients with peaking BKV were lymphopenic and were undergoing triple-drug immunosuppressive therapy (tacrolimus, mycophenolate mofetil, and prednisone) at the time of BKV diagnosis. Resolving samples were collected following the net reduction of immunosuppression after the diagnosis of BK viremia or nephropathy. Clinical data were extracted from electronic medical records. Kidney histopathology slides from the biopsy were evaluated by an expert renal transplant pathologist to confirm the diagnosis of BKVN when SV40 or other histological features suggestive of BKVN were present, including hallmark viral cytopathic effects. Banff 2022 scoring was performed on available histological samples at the time of biopsy (Table 3) (51). Using a model that integrates Banff scoring to predict the severity of inflammation and rejection, we observed that all the biopsies from allPeak and resBKVir groups had low acute lesion scores (52). Healthy kidney allografts in the first year after transplant (control samples, *n* = 12) were described previously in the paper by Menon et al. (26).

### Single-cell processing of research biopsy cores.

The procedures for tissue processing, single-cell isolation, and scRNA-seq have been previously detailed in earlier publications(53). We adhered to the official standardized operating procedure outlined by the Kidney Precision Medicine Project (KPMP) (https://www.protocols.io/view/single-cell-RNA-seq-scrna-seq-7dthi6n). Briefly, kidney biopsies were stored in CryoStor and frozen in liquid nitrogen until required. The vials were rapidly thawed, and the biopsies were subsequently dissociated into a single-cell solution. Specifically, biopsy tissue fragments of 3–5 mm (16-gauge needle) were enzymatically digested using Liberase TL (Roche) for 12 minutes at 37°C. The solution was then filtered through a 30 μm strainer (Miltenyi Biotec) and washed in DMEM/F12 medium, supplemented with 10% fetal calf serum. Next, 20,000 viable cells were processed utilizing the droplet-based 10x-Genomics platform with Chromium Single Cell 3’ chemistry (v3.1). Following cDNA library preparation, sequencing was conducted on an Illumina NovaSeq 6000 platform, generating 200 million paired-end reads (2 × 151 bases) per sample. scRNA-seq reads were processed using Cell Ranger v7.0.1(54).

Reads were aligned to a combined reference genome that included the human reference genome (GRCh38) and the BK polyomavirus genome (NC_001538) to enable the identification of both human and BKV transcripts. To visualize read coverage, alignment quality, and variant distribution, BAM files were loaded into Integrated Genomics Viewer (IGV). The coverage track was used to assess read depth at each genomic position, while IGV’s color-coded variant display provided insights into variant frequency and sample specificity. BKV gene annotations were overlaid to contextualize observed variants within the viral genome. Patient-specific variants were identified by comparing variant profiles between samples, ensuring that differences were not due to sequencing artifacts or contamination.

Seurat v4.0.4 R package was used for downstream analysis (55). The following quality control preprocessing measures were implemented on the combined object containing the scRNA-seq data across all samples: ambient RNA detection and correction using SoupX with default parameters, filtering out cells with fewer than 500 or more than 5,000 genes with reads, and removing cells with greater than 50% reads mapping to mitochondrial genes (56). The data were log-normalized, and sample integration was performed using the CCA-derived integration method within Seurat (55). For dimensionality reduction and visualization, principal component analysis (PCA) was run on the log-normalized counts, and the first 20 principal components (PCs) were selected. Using the Euclidean distance metric, we implemented the standard procedure in Seurat to construct cell type clusters: the K-Nearest Neighbor (kNN) graph was built for k = 20 nearest neighbors. The kNN graph was then used to construct a shared-nearest neighbor (sNN) graph by calculating the neighborhood overlap between every cell and its 20 nearest neighbors using the Jaccard index. Louvain clustering was applied to the sNN graph with resolution = 0.5 and otherwise default parameters. Clusters were annotated by differential expression analysis of genes expressed in each cluster versus all other clusters (FDR < 0.05) and known kidney-specific marker genes (PREMIERE and KPMP kidney cell integration atlas; Supplemental Table 1) (57). Subclustering with resolution 0.03 revealed separate T cell and podocyte clusters. Doublet detection was considered using DoubletFinder (58); however, it was ultimately not included in the analysis as the identified doublets were very few and did not consistently converge between runs, offering no additional benefit to downstream analyses. For visualization (Figure 1 and Supplemental Figure 1), the sNN graph capturing the underlying topology of the expression data was then subject to uniform manifold approximation and projection (UMAP).

### Cell line comparison.

The same quality control preprocessing measures for the biopsy single cell data were implemented on the cell line data from An et al. (23). The An et al. dataset is a scRNA-seq dataset of BK-virus–infected renal proximal tubule epithelial cells in vitro at 2 time points after infection: day 2 (d2) and d5. After quality control filtering, this dataset comprised 4,558 d2 mock cells; 3,996 d2 BKV-inoculated cells; 2,839 d5 mock cells; and 3,382 d5 BKV-inoculated cells. Differentially expressed genes (DEGs) were detected between d2-infected and mock and d5-infected and mock at each of the 2 time points using the Seurat FindMarkers function.

### Functional literature-based analyses.

Module detection is a community clustering approach that identifies groups of genes tightly connected within a biological network (Supplemental Figure 2) (27–29). In this study, module detection leveraged relevant tissue-specific networks constructed via a Bayesian integration framework (including proximal tubule, T-lymphocyte, macrophage, monocyte, and NKC-specific networks) (28). These networks encode the likelihood of functional interactions between genes within a specific tissue context by combining large-scale genomic datasets and prior biological knowledge. Module detection using these networks identifies tissue-specific gene relationships, which may not be captured by global, tissue-agnostic networks or traditional GO enrichment analysis. This network-based method enables the discovery of biologically significant gene modules, even when not annotated in existing databases. Furthermore, it even assigns genes without canonical pathway annotations to modules, which can aid in identifying additional genes that function in a process or pathway.

Kidney-specific functional analyses were performed using HumanBase (https://hb.flatironinstitute.org/) (27–29). For each cell type and system of interest, module detection was performed using all DEGs between peaking and SURV states or between peaking and resolving phases of infection (FDR < 0.05). GO Term enrichment analysis on all modules identified functional processes within each module. This 2-step approach (network-based module detection followed by GO enrichment) ensures that biologically meaningful pathways are captured, including those involving a subset of DEGs or genes not traditionally associated with canonical processes.

### Cell cycle scoring.

The “CellCycleScoring” utility function in Seurat was used to score each cell in the scRNA-seq data based on canonical markers to predict the “G2M,” “S,” or “G1” phase of the cell cycle. The scores were then compared between BKV-infected and control biopsies.

### IHC.

After antigen retrieval and blocking, FFPE sections were dual-stained for SV40 (MRQ-4, Cell-Marque) or VP1 (M22/6E10, Abnova) with CD45 (PD7, Thermo). Goat anti-mouse isotype-specific secondary antibodies linked to horseradish peroxidase and alkaline phosphatase (Southern Biotechnology) were used, followed by Betazoid DAB or Ferangi Blue (Biocare).

### Electron microscopy.

Kidney tissues were fixed in 3% formaldehyde/glutaraldehyde with 2% osmium tetroxide and impregnated with uranyl acetate. After embedding in Epon 812, thin sections were cut and stained with lead citrate. Images were captured using a BX41 scope with a DP73 camera (Olympus) or an HT7500 (Hitachi).

### Statistics.

Differential expression analysis was performed using a nonparametric Wilcoxon rank-sum test, with *P* values adjusted by Bonferroni’s correction. Gene ontology enrichment analysis was performed using a 1-sided Fisher’s exact test, with correction for multiple hypothesis testing using the Benjamini-Hochberg method. Fisher’s exact test was also used to assess enrichment of TCMR-associated genes within the BKV transcriptomic signature. Cycling cell proportion comparisons were performed using a 1-tailed Welch’s 2-sample *t* test. A cutoff of 0.05 was used to assess statistical significance.

### Study approval.

The University of Michigan IRB approved this study. Biopsy tissues were obtained from willing participants who provided written, informed consent (IRB HUM0015096). All clinical and research activities reported here are consistent with the principles of the Declaration of Istanbul.

### Data availability.

All expression data are deposited in the Gene Expression Omnibus and can be accessed using the accession no. GSE317012. Supplemental Tables in XLS format are present in the file “Formatted_compiled_Supplemental_tables.” Supporting Data Values provides data behind the main figures.

## Author contributions

TM, RS, and PJM are all designated co–first authors. All played independent and critical roles in this study. TM wrote the initial draft of the manuscript with ASN and did the preliminary analysis. RS performed several analysis including genotype analysis. PJM was instrumental in initial processing of the scRNA-seq and subsequent analysis. ASN obtained funding for the biorepository used to generate underlying data. KJS obtained the clinical data associated with biopsy samples. RG, ASN, and SPN contributed to the tissue samples in the biorepository. TM, RS, PJM, FA, LS, TJR, CCB, EAF, FE, P An, PGC, and JMP analyzed the data. PGC performed the bioinformatic analysis of BKV sequences, including inspection of read alignments in IGV and variant/SNP analysis confirming that each infected biopsy carried a single, unique BKV strain. P Arrowsmith was responsible for immunostaining of the biopsies. TM, RS, PJM, FE, EAF, MJI, JMP, OGT, EAO, RM, MK, CLT, and ASN interpreted the data. EAO was responsible for single-cell preparation in the initial study by Menon et al. (26) that provided the single-cell transcriptomic data. TM, RS, PJM, CLT, and ASN wrote the manuscript with critical input from MJI, OGT, and MK. All authors agree to the final version of this manuscript and consented to submission.

## Conflict of Interest

ASN discloses a filed patent “TARGETING THE GH-IGF-1 PATHWAY TO EXTEND ORGAN LIFESPAN”; U.S. Provisional Patent Appl. No.: 63/460,140 unrelated to this work.

## Funding support

This work is the result of NIH funding, in whole or in part, and is subject to the NIH Public Access Policy. Through acceptance of this federal funding, the NIH has been given a right to make the work publicly available in PubMed Central.

- National Institutes of Health T32HG003284 (TM)
- AI060584 (MJI)
- RO1AI153156 (JMP)
- National Institutes of Health grant R01GM071966, U24DK100845, U01DK133090, U01DK114907, and Simons Foundation grant 395506 (OGT)
- George M. O’Brien Michigan Kidney National Resource Center, funded by NIH/NIDDK grant U54DK137314 (MK)
- George M. O’Brien Michigan Kidney National Resource Center, funded by NIH/NIDDK grant U54DK137314, the First Pathway Award, Michigan Institute of Clinical Health Research UL1TR002240, Department of Internal Medicine, and the National Institute of Health K23 DK 125529 (ASN)

## Supplementary Material

Supplemental data

ICMJE disclosure forms

Supplemental tables 1-14

Supporting data values

## Figures and Tables

**Table 1:**
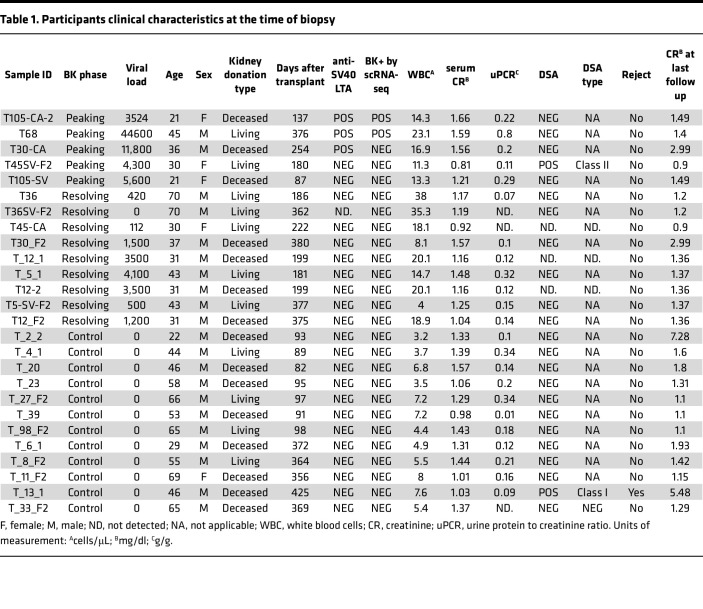
Participants clinical characteristics at the time of biopsy

**Table 2:**
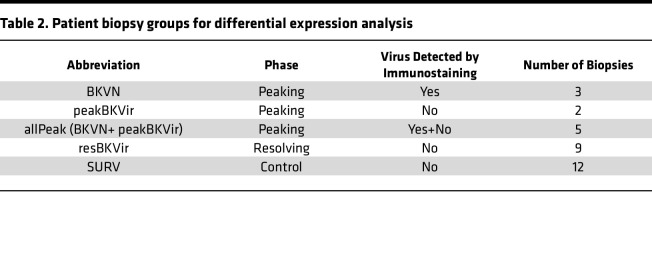
Patient biopsy groups for differential expression analysis

**Table 3:**
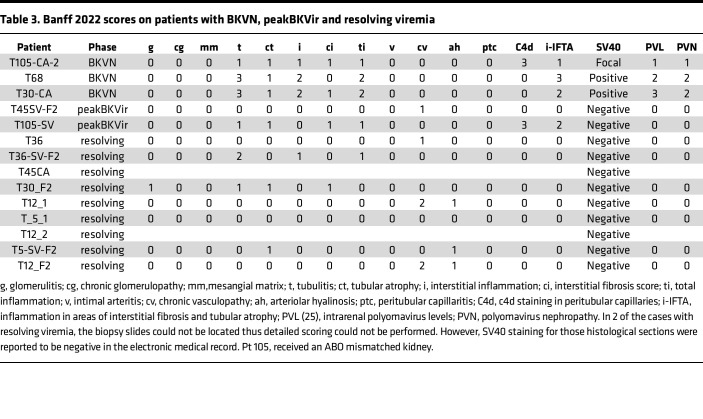
Banff 2022 scores on patients with BKVN, peakBKVir and resolving viremia

**Table 4:**
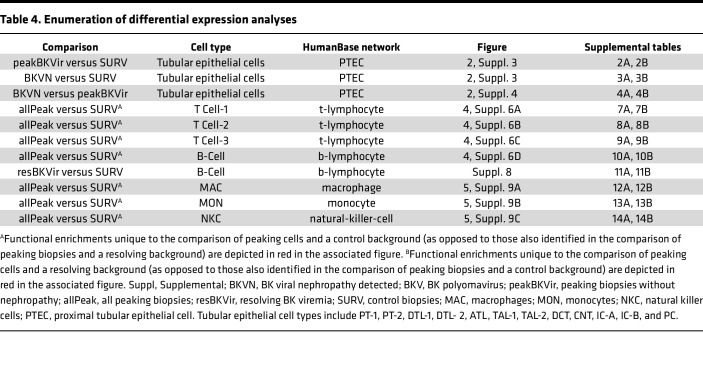
Enumeration of differential expression analyses

## Version 1 03/12/2026

In-Press Preview

## Version 2 04/22/2026

Electronic publication

## Version 3 07/17/2026

Paul G. Cantalupo added to author list and PGC added to Author contributions section
