# Supplementary material for: Single-cell profiling reveals epithelial and immune responses in BK polyomavirus–infected human kidney biopsies
Source: JCI Insight. 2026 Mar 12;11(8):e198227. doi: 10.1172/jci.insight.198227 (PMC13135396; doi:10.1172/jci.insight.198227)

Supplementary Figure S1: Single Cell RNA-seq analysis of biopsies
UMAPs for cells from each category of biopsy, Peaking (allPeak; Groups 2+3), Resolving (resBKVir; Group 4), and Surveillance (SURV; Group 1). Each biopsy category contributes cells to each of the 30 clusters. Cell-type abbreviations are consistent with Figure 1.

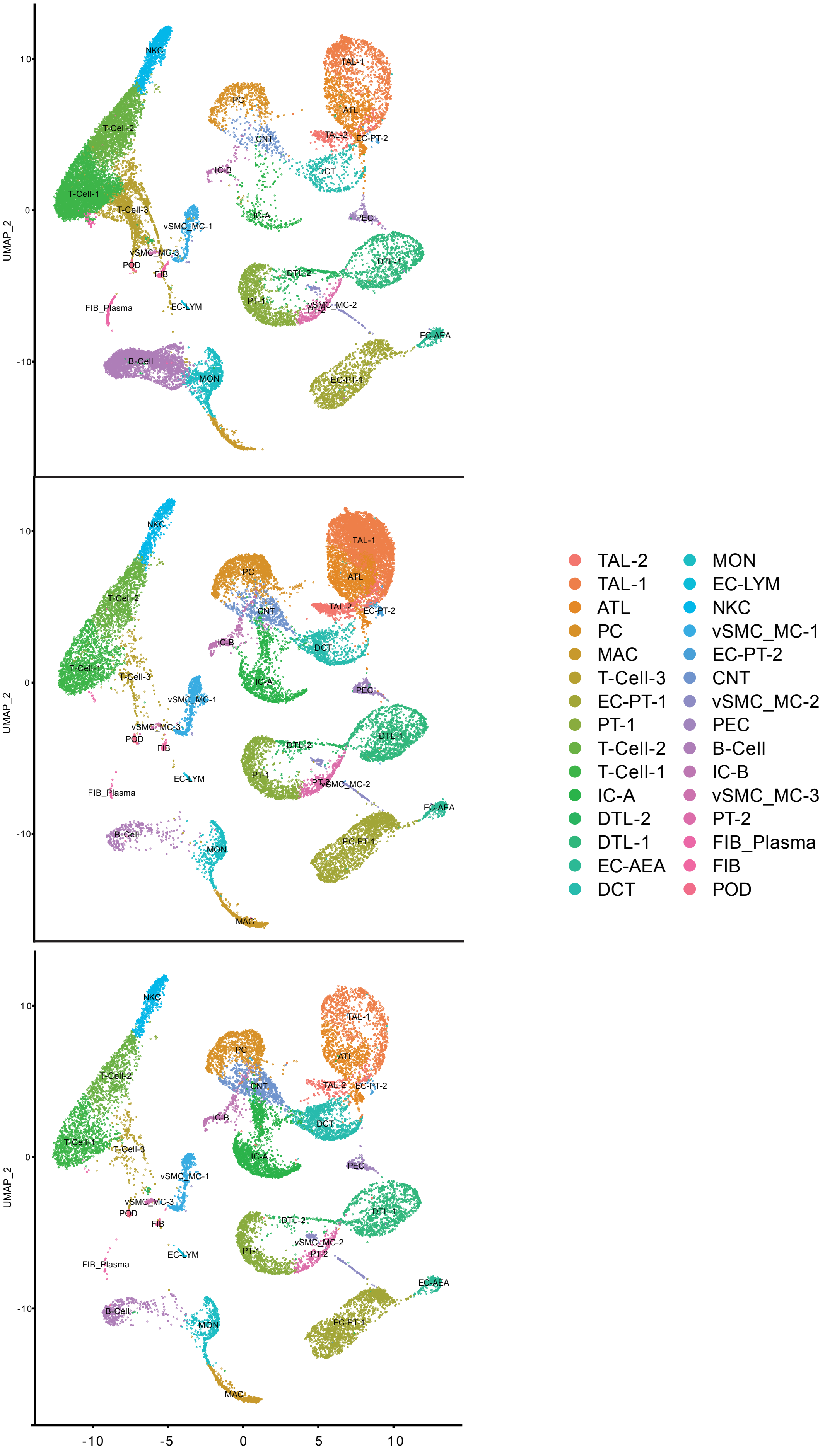

Supplementary Figure S2. Workflow for functional module detection in tissue-specific gene interaction networks.

The process begins with a cell-type specific gene interaction network from the GIANT database. These networks are built using a Bayesian framework that integrates diverse genomic datasets, including tissue-specific gene co-expression, protein-protein interactions, functional genomic screens, transcription factor binding data, comparative genomics, and curated literature. Edge weights in the GIANT network represent the probabilistic likelihood of a functional relationship between genes in the given tissue. Thicker lines indicate more probable functional interactions. A subnetwork is extracted by parsing the full GIANT network to include only the user-provided differentially expressed genes. Edges in the subnetwork are reweighted based on the number of shared k-nearest neighbors (SKNN), emphasizing local network structure and reducing the influence of high-degree hub nodes. The filtered subnetwork is clustered into distinct functional modules using the Louvain algorithm, run iteratively to calculate comembership scores for gene pairs. Resulting modules represent cohesive groups of genes that are tested for enrichment in Gene Ontology (GO) biological processes, providing biological context for each module.

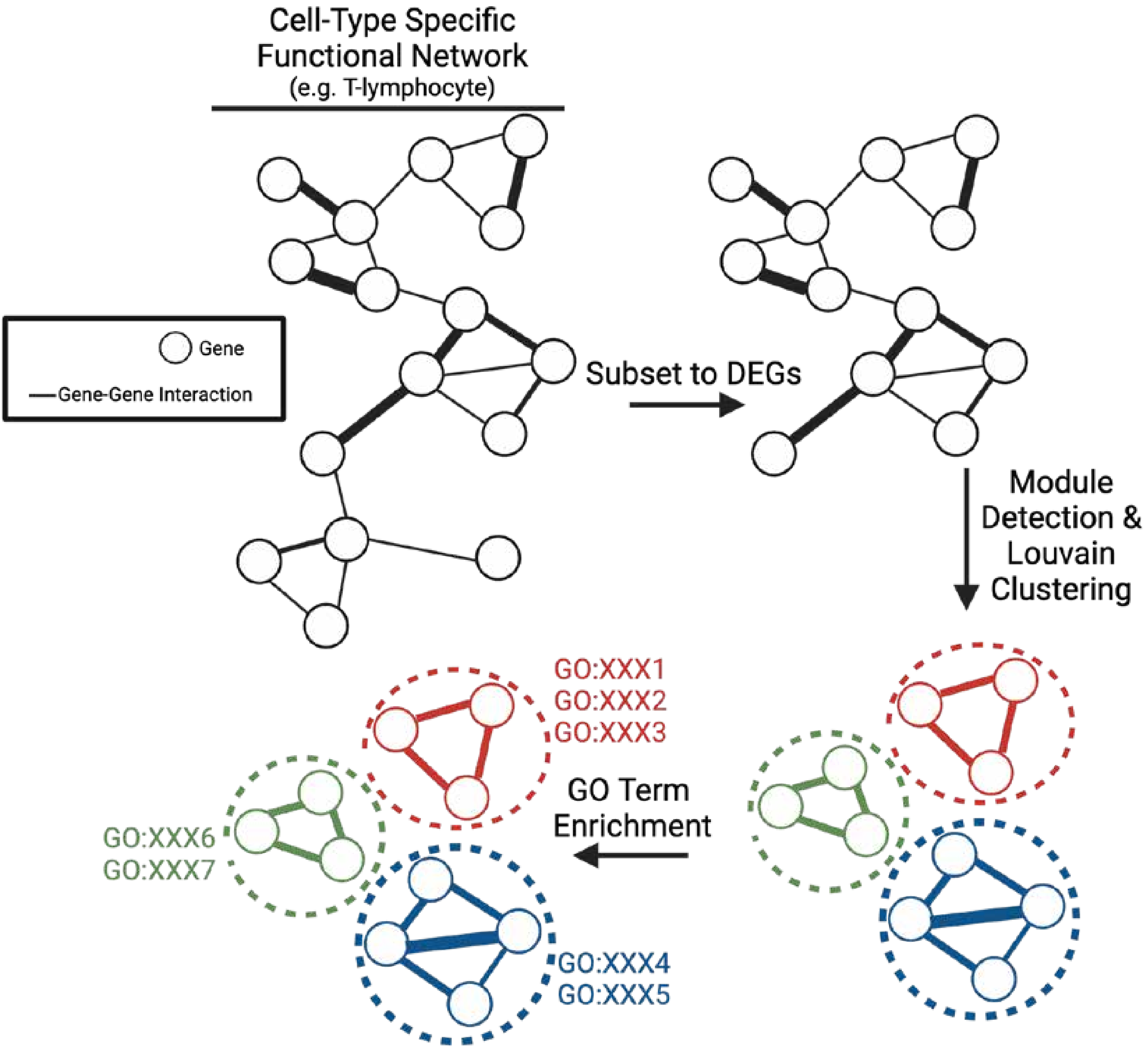

Supplementary Figure S3: Functional Network Enrichment - Expression signature of tubular epithelial cells with BKVN compared to surveillance biopsies.

The transcriptomic signatures of tubular epithelial cells in peaking BKV (allPeak; Groups 2+3) were compared to the expression profiles of tubular epithelial cells from controls (SURV; Group 1). Functional enrichments unique to BKVN biopsies (Group 3 as opposed to those also identified in tubular epithelial cells from peakBKVir biopsies/Group 2) are depicted in red. Genes upregulated in the BKVN signature (Group 3) were enriched for polyomavirus infection hallmarks, including viral entry into host cells, inflammation, ribosome biogenesis, translation, energy restructuring, and cell cycling (M1, M3, M6). BKVN also initiated a robust innate immune response (M2)—peakBKVir cells induced signatures related to stress signaling, apoptosis, and energy remodeling. Nephropathy induces cytokine signaling, translation, regulation of cell-cycle signaling, and wound healing (red font).

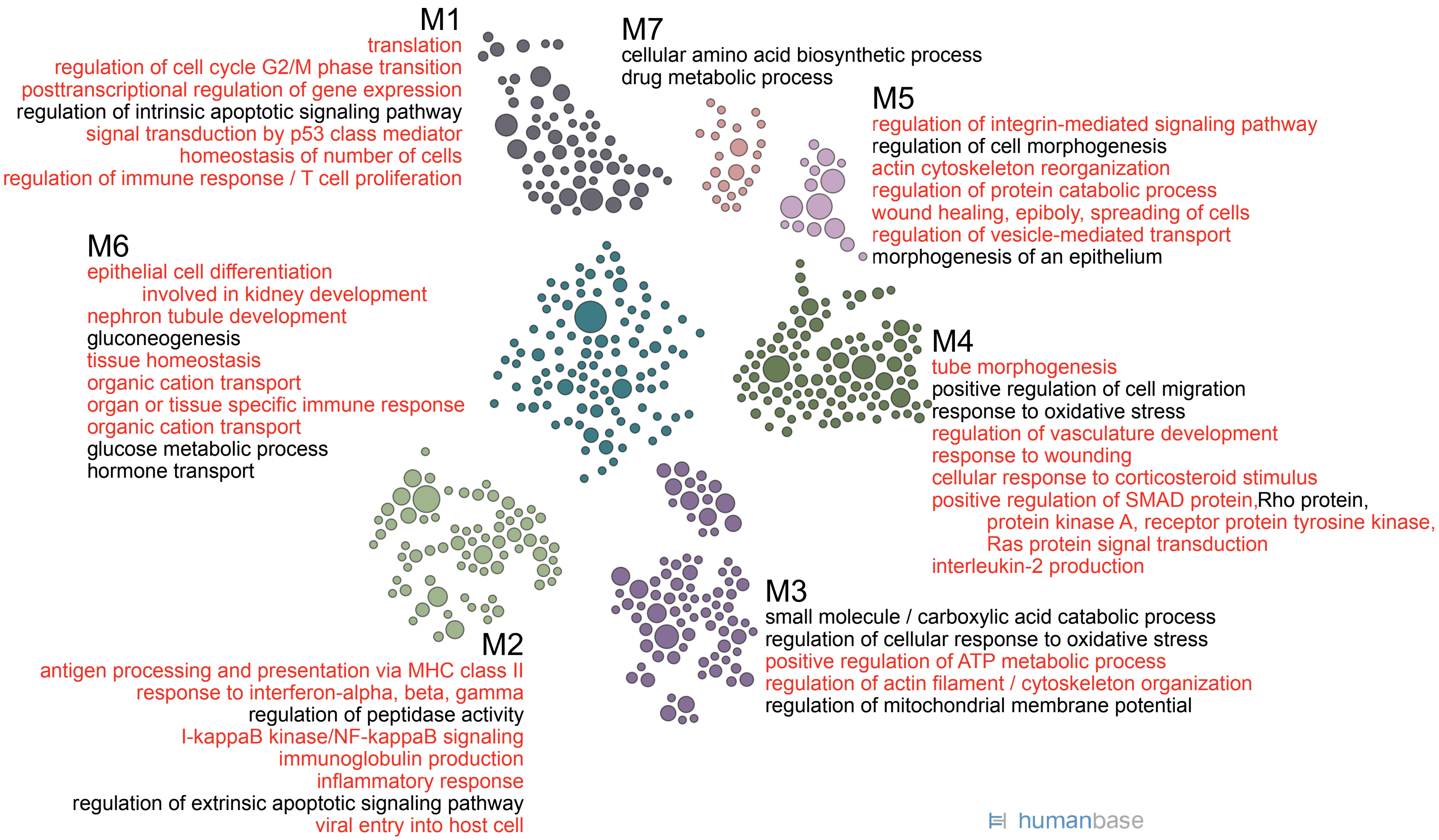

Supplementary Figure S3: Functional Network Enrichment - Expression signature of tubular epithelial cells with BKVN compared to surveillance biopsies.

The transcriptomic signatures of tubular epithelial cells in peaking BKV (allPeak; Groups 2+3) were compared to the expression profiles of tubular epithelial cells from controls (SURV; Group 1). Functional enrichments unique to BKVN biopsies (Group 3 as opposed to those also identified in tubular epithelial cells from peakBKVir biopsies/Group 2) are depicted in red. Genes upregulated in the BKVN signature (Group 3) were enriched for polyomavirus infection hallmarks, including viral entry into host cells, inflammation, ribosome biogenesis, translation, energy restructuring, and cell cycling (M1, M3, M6). BKVN also initiated a robust innate immune response (M2)—peakBKVir cells induced signatures related to stress signaling, apoptosis, and energy remodeling. Nephropathy induces cytokine signaling, translation, regulation of cell-cycle signaling, and wound healing (red font).

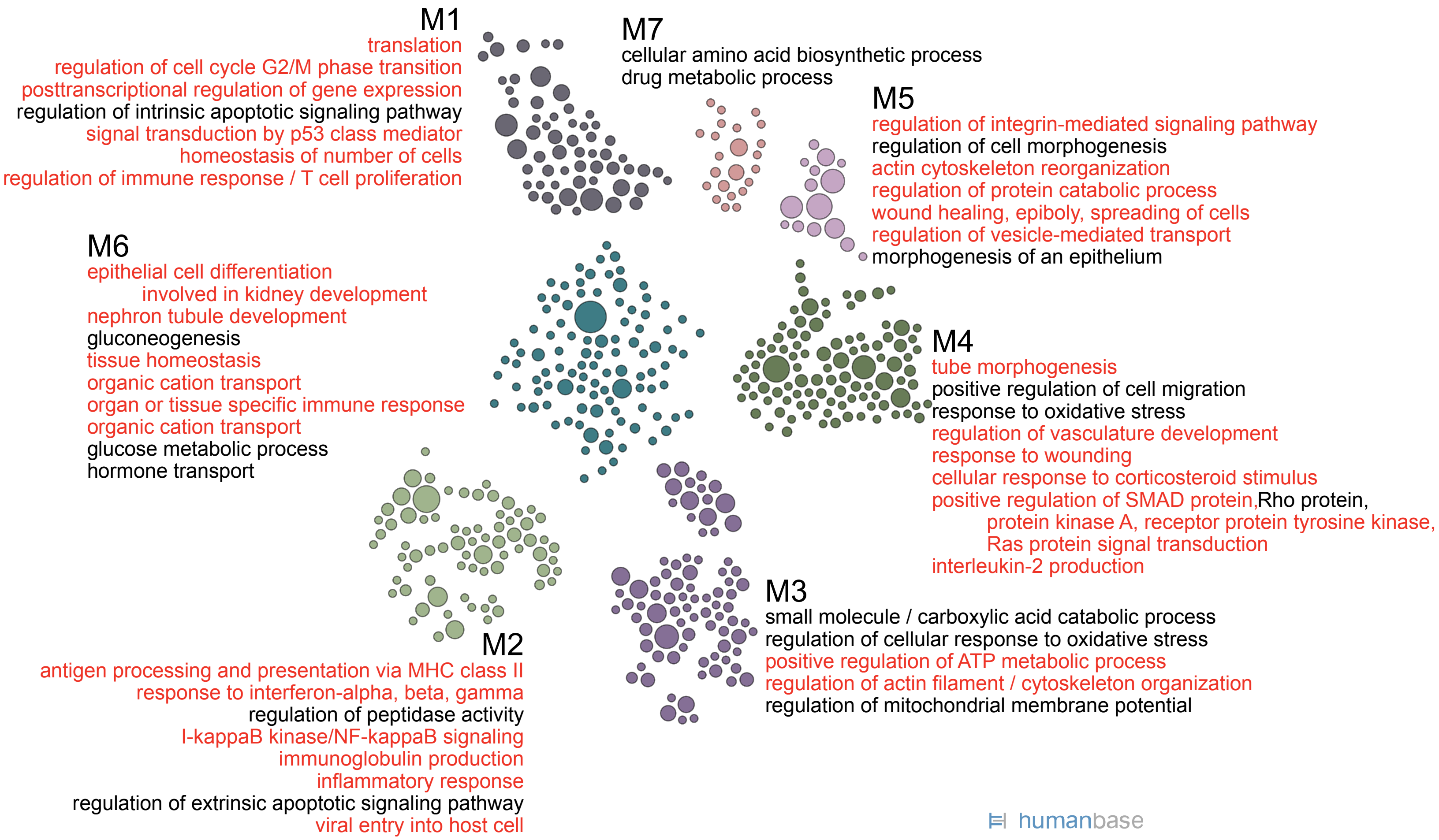

Supplementary Figure S4: Functional Network Enrichment - Comparing the expression signatures of BKVN and peakBKVir tubular epithelial cells.

When directly comparing epithelial cells in three biopsies with BKVN (Group 3) to peakBKVir biopsies (Group 2), we observed that patients with BKVN had increased expression of genes associated with antigen processing and presentation and interferon stimulated genes. We also note that tubular epithelial cells from BKVN patients exhibit upregulation of pathways involved in wound healing, extracellular matrix remodeling, and protein biosynthesis, suggesting an epithelial response to injury and repair.

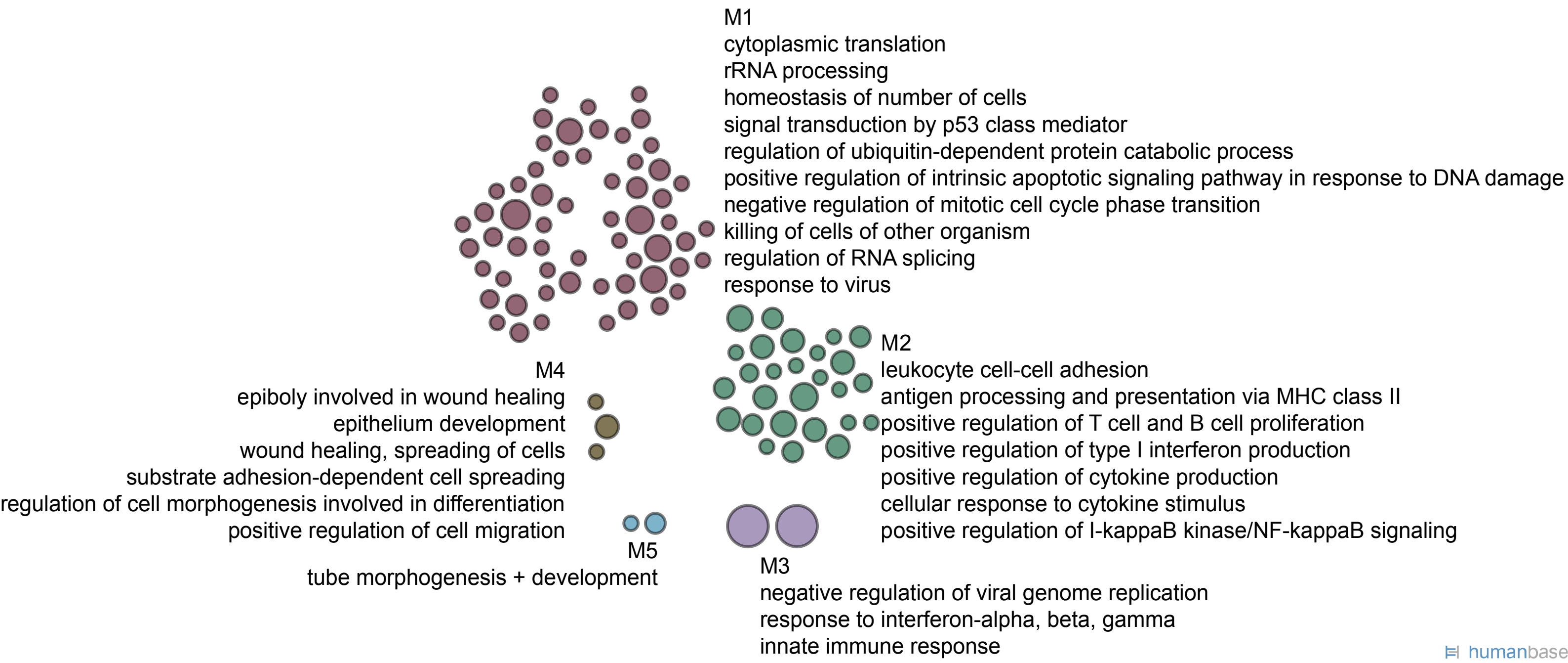

Supplemental Figure S5: Assessing the role BK infection plays in regulating cell cycling.
The highest proportion of adaptive immune cells with G2/M or S cell cycle signal score was observed in peaking biopsies compared to those from surveillance and resolving samples, indicating the cells in peaking samples are preferentially in G2/M or or S. * indicates pval <0.05, *** indicates pval <0.001.

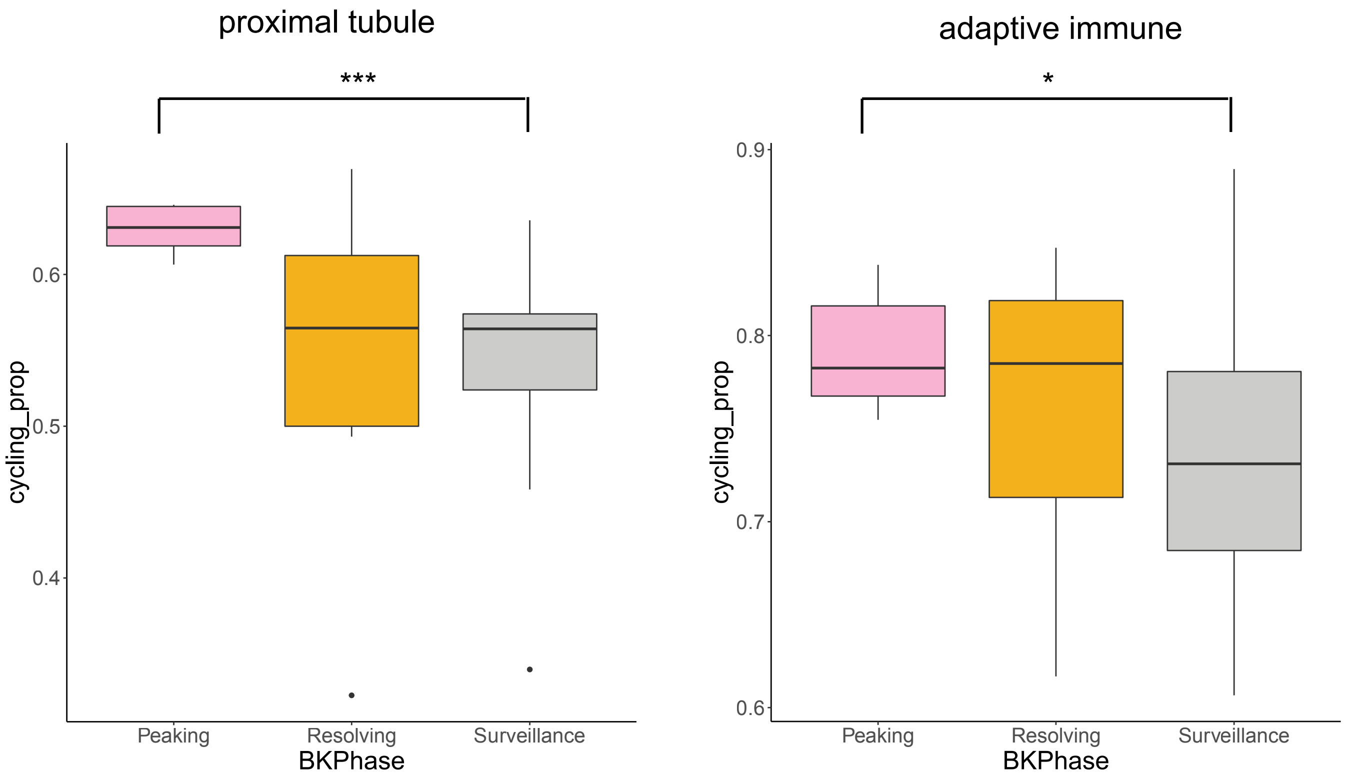

P(Peaking > Surveillance) = 0.00084

P(Peaking > Surveillance) = 0.023

Supplementary Figure S6: Functional Network Enrichment - Expression signatures from T and B cells, comparing allPeak to surveillance biopsies.

Functional enrichments unique to the comparison of allPeak (Groups 2+3) and SURV (Group 1) (as opposed to those enrichments also identified in the comparison of allPeak and resBKVir/Group 4) are depicted in red. The t-lymphocyte network-based functional enrichment of DEGs between T cells derived from allPeak and SURV biopsies was performed using HumanBase.io. Processes activated in all T-cell clusters included lymphocyte proliferation and migration, protein translation, and antiviral interferon signaling. A. Functional enrichment of the T-Cell-1 (expressing Th17 markers) allPeak expression signature versus the T-Cell-1 SURV expression signature. T-Cell-1 allPeak cluster exhibits an upregulation of cytokine production, cellular respiration and energy metabolism, RNA splicing activity, and signal transduction cascades. B. Functional enrichment of the T-Cell-2 allPeak expression signature versus the T-Cell-2 SURV expression signature. Peaking cells from T-Cell-2 express markers for CD8+ T cell marker genes and are enriched for genes related to the cellular response to cytokine stimulus. Pathways related to T cell-mediated cytotoxicity are upregulated. C. Functional enrichment of the T-Cell-3 (expressing Treg markers) allPeak expression signature versus the T-Cell-3 SURV expression signature. Upregulation of protein ubiquitination pathways suggest post-translational regulation of key signaling molecules. D. Functional enrichment of the B cell allPeak expression signature versus the B cell SURV expression signature. Enriched processes in allPeak B cells included antigen processing and presentation, translation, leukocyte activation and proliferation, and several stimulated signaling pathways.

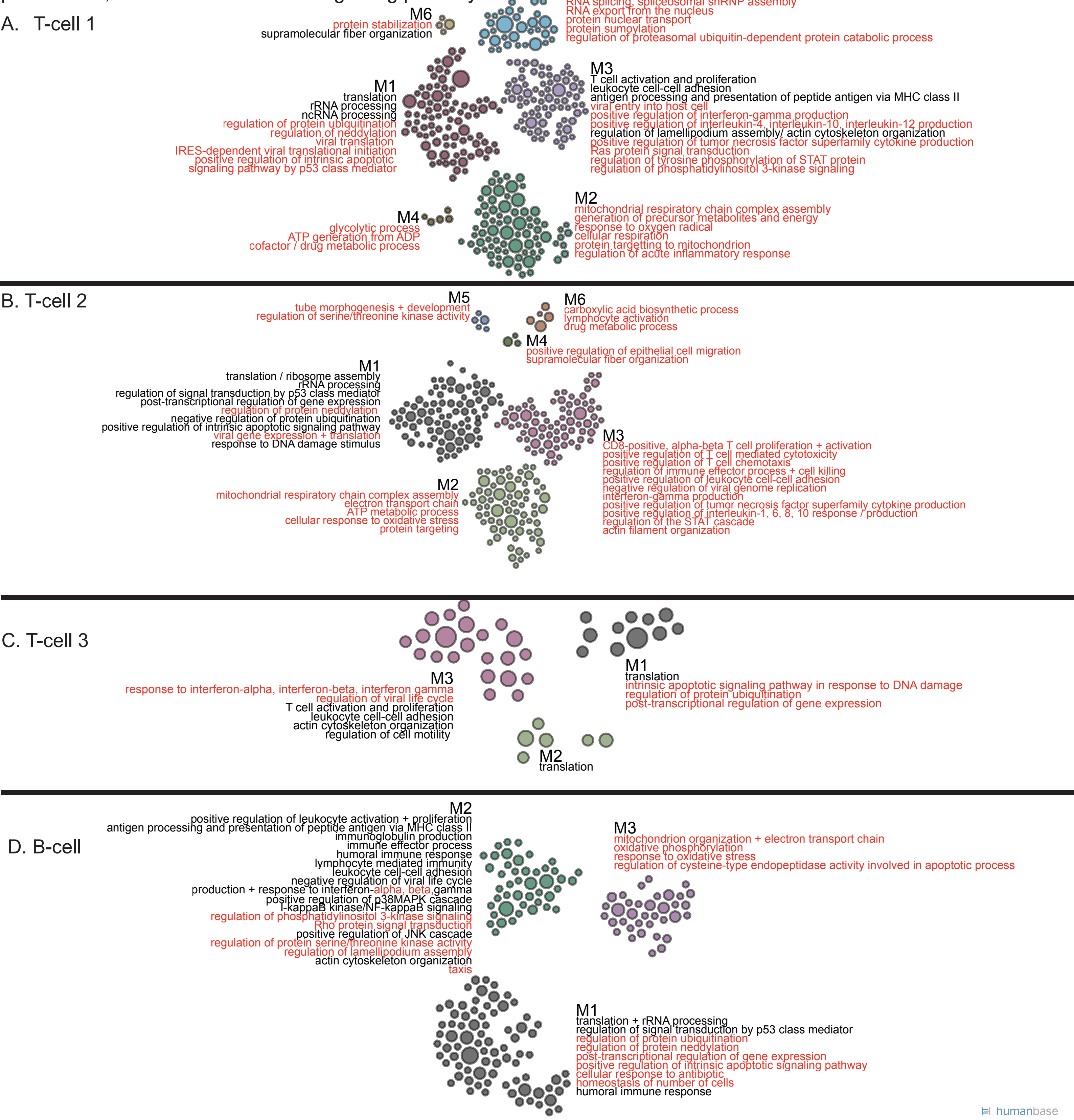

Supplementary Figure S7: Cell-type annotation of the three T Cell Clusters using known immune marker genes.

Gene expression for canonical immune cell marker genes for all cells contained in the three T cell clusters (which included cells from peaking, resolving, and surveillance biopsies).

(A) Expression of genes commonly expressed across multiple T cell subsets.

(B) Expression of selected genes that distinguish major T cell subtypes, including CD8+ T cells (red annotation), T helper 17 cells (Th17, purple annotation), and regulatory T cells (Tregs, brown annotation). Expression patterns suggest T-Cell-1 cells are Th17 cells, T-Cell-2 cells are CD8+ cytotoxic T cells, and T-Cell-3 cells are Tregs.

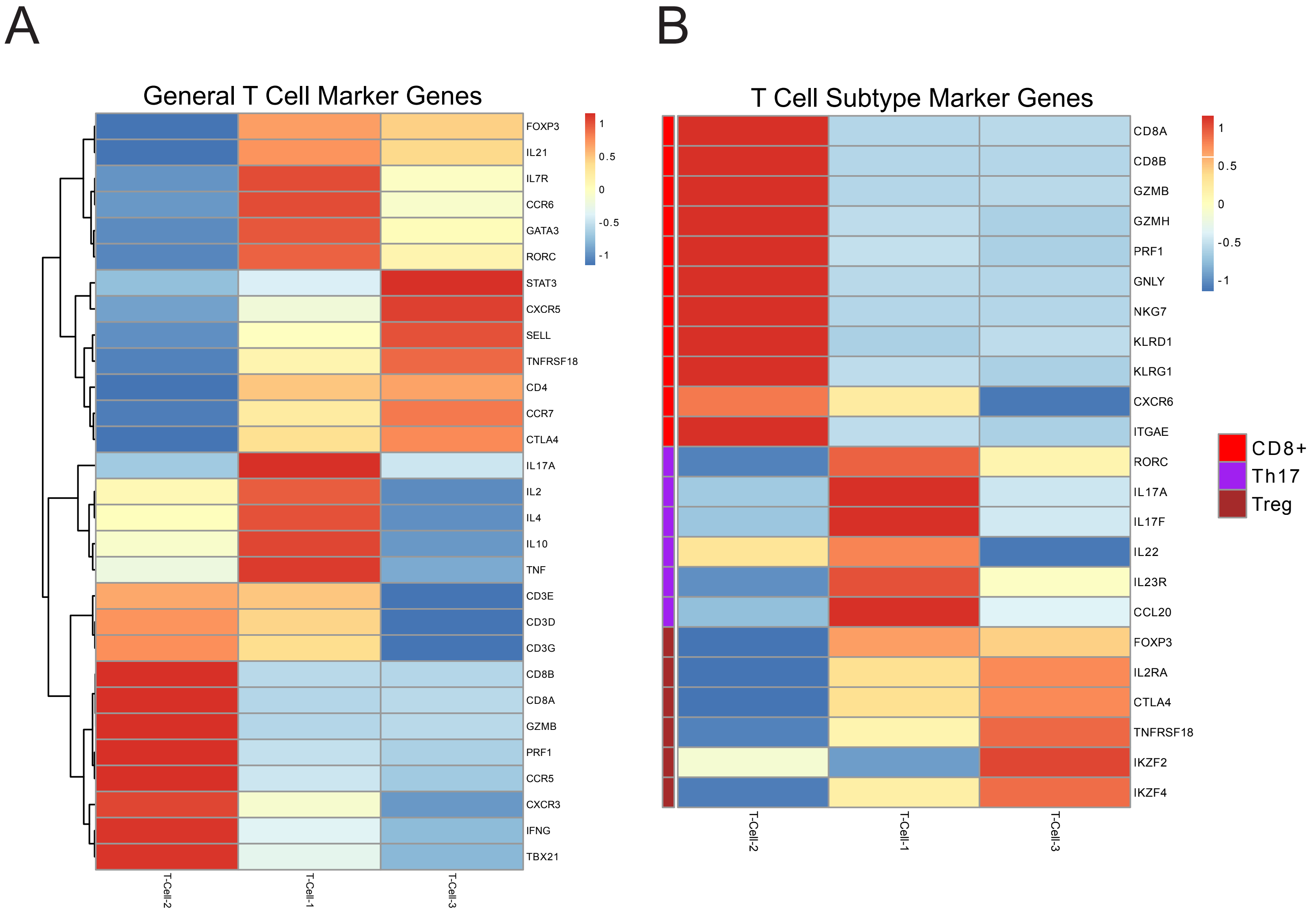

Supplementary Figure S8: Persistent Immune Activation and Apoptotic Signatures in B Cells During Resolving BK Viral Infection

Direct comparison of B cells from resBKVir (Group 4) to SURV (Group 1) revealed signatures of viral defense and immune activation. Despite entering the resolving phase, B cells maintain upregulation of genes involved in ribosome biogenesis, leukocyte activation, and apoptosis, suggesting ongoing protein synthesis, antigen presentation, and immune signaling. The persistence of apoptotic gene expression indicates continued B-cell turnover, raising the question of whether this profile could contribute to the development of donor-specific antibodies. Statistical significance of each fold change is denoted by * = adjusted p-value < 0.05, ** = adjusted p-value < 0.01, *** = adjusted p-value < 0.001.

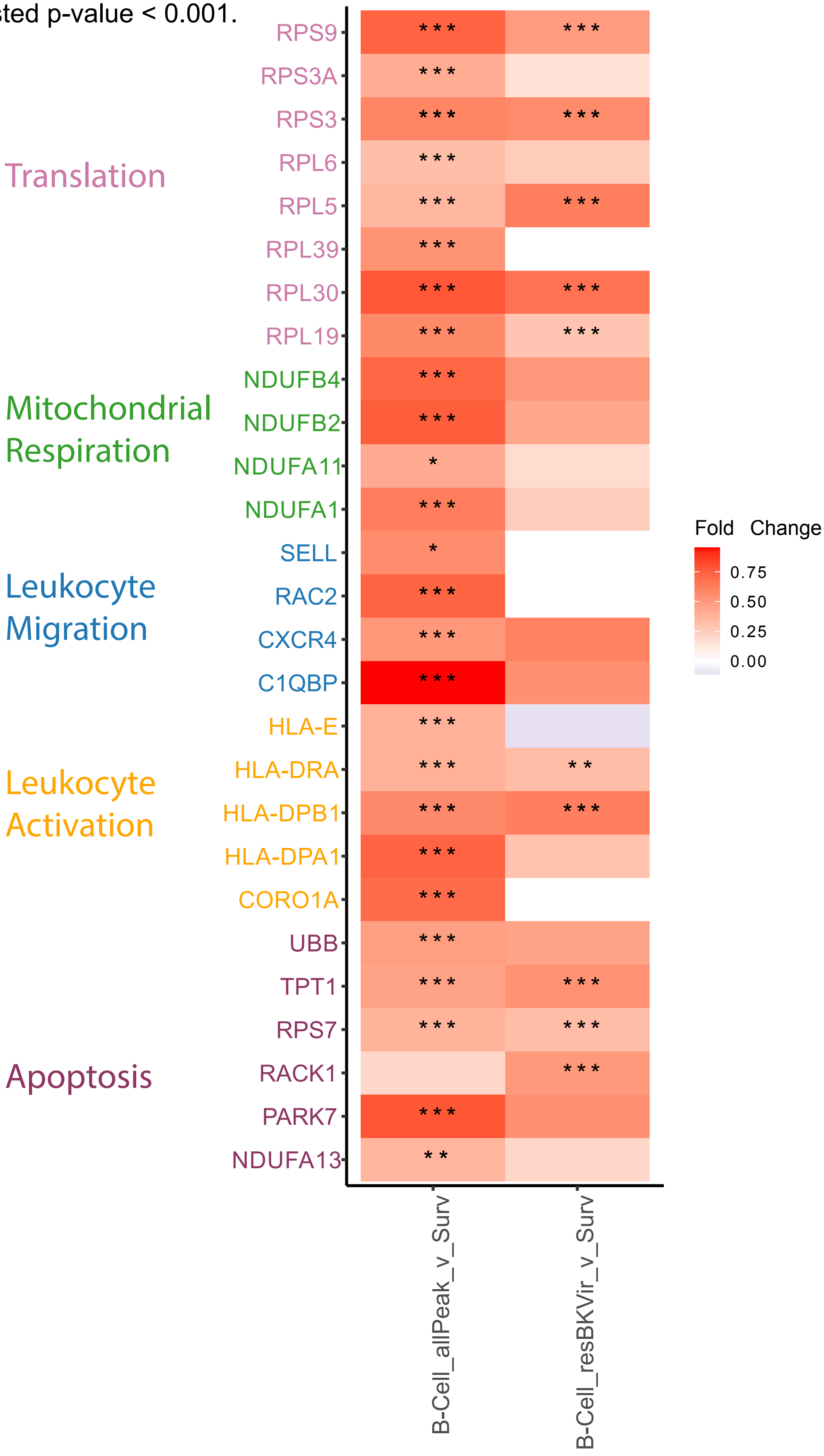

Supplementary Figure S9: Functional Network Enrichment - Innate immune cells responses with peaking BK viremia compared to surveillance biopsies.

Functional enrichments unique to the comparison of allPeak (Groups 2+3) and SURV (Group 1) (as opposed to those also identified in the comparison of allPeak and resBKVir/Group 4) are depicted in red. Genes associated with translation, RNA metabolic processes, and response to the virus were upregulated across innate immune cell types in allPeak compared to SURV biopsies. A. Functional enrichment of genes upregulated in allPeak v. SURV macrophages B. Functional enrichment of genes upregulated in allPeak v. SURV monocytes C. Functional enrichment of genes upregulated in allPeak v SURV natural killer cells included positive regulation of lymphocyte proliferation, antigen processing and presentation, response to interferon-gamma, response to cytokine stimuli, and response to virus.

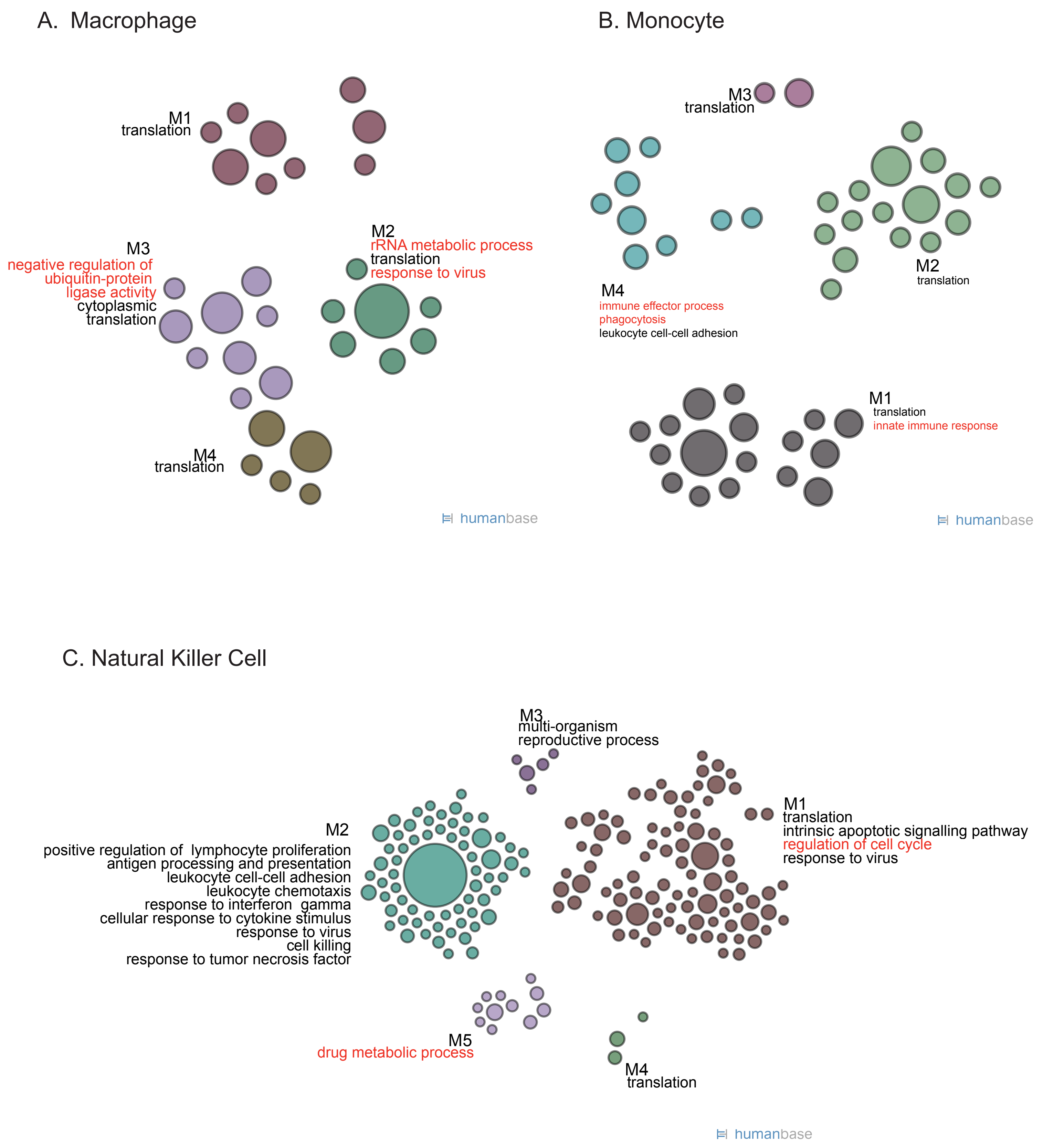

Supplementary Figure S10: Enhanced Translational Activity and Cytoskeletal Remodeling in Innate Immune Cells During BK Virus Nephropathy

Direct comparison of innate immune cells from BKVN (Group 3) to peak BK viremia (Group 2) revealed increased translational activity across macrophages (MAC), monocytes (MON), and natural killer cells (NKC). BKVN was associated with upregulation of ribosomal genes, suggesting enhanced protein synthesis, as well as differential expression of cytoskeletal regulators (ACTB and CFL1), indicating potential shifts in immune cell structure and function. Statistical significance of each fold change is denoted by * = adjusted p-value < 0.05, ** = adjusted p-value < 0.01, *** = adjusted p-value < 0.001.

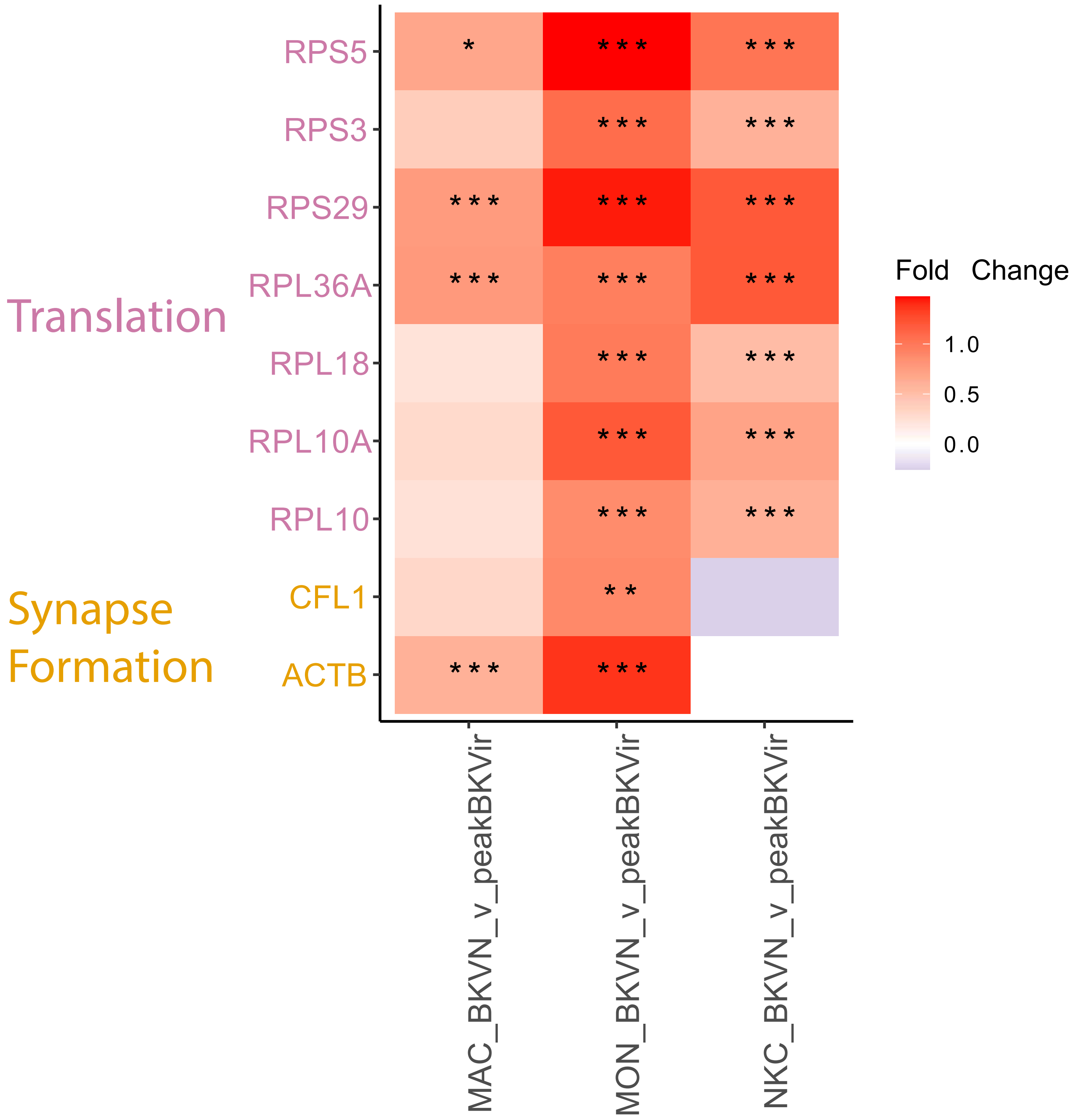

Supplementary Figure S11: BK virus transcriptional reads and viral proteins are detectable in patients.

A. Cell identity and cell counts for BKV transcripts were detected in two biopsies (samples T68 and T105-CA-2). To account for differences in total cell recovery across cell type clusters, the number of BKV transcript-positive cells was normalized to the total number of cells identified within each cluster. Bar plots below show the proportion of transcript-positive cells across different cell type clusters for each viral transcript. **B.** Immunohistochemistry (IHC) staining of BK viral proteins. Left panel: Large T Antigen (brown) shows nuclear staining restricted to tubular epithelial cells (filled arrow), distinct from interstitial CD45+ leukocytes (blue). Right panel: VP1 (brown) is similarly positive in tubular epithelial cells (filled arrow). At the same time, most CD45+ leukocytes (blue) are interstitial, but there are rare cells within clusters of leukocytes that show co-staining for both CD45+ and VP1 (open arrow). The image is 400x.

A.

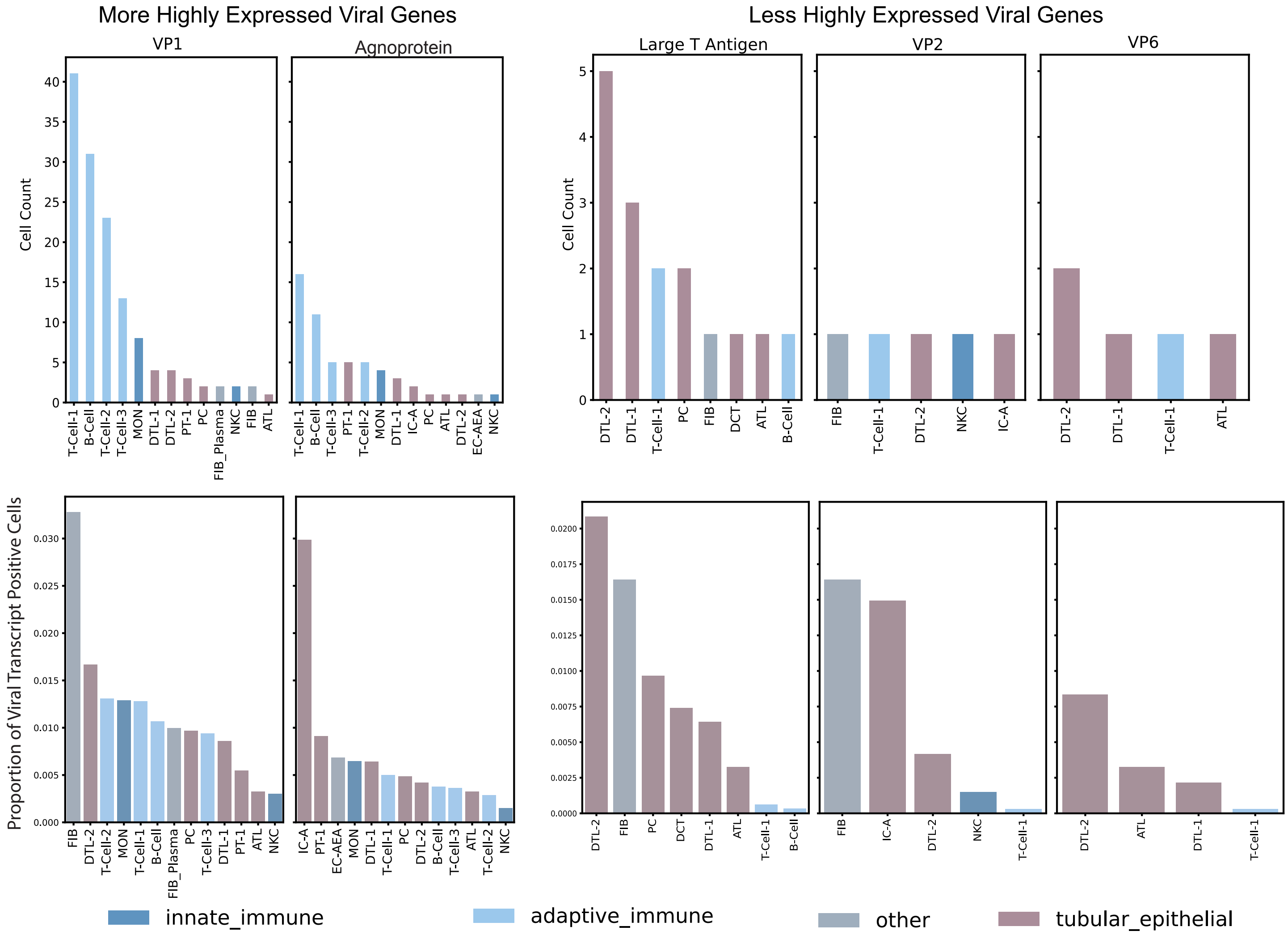

B.

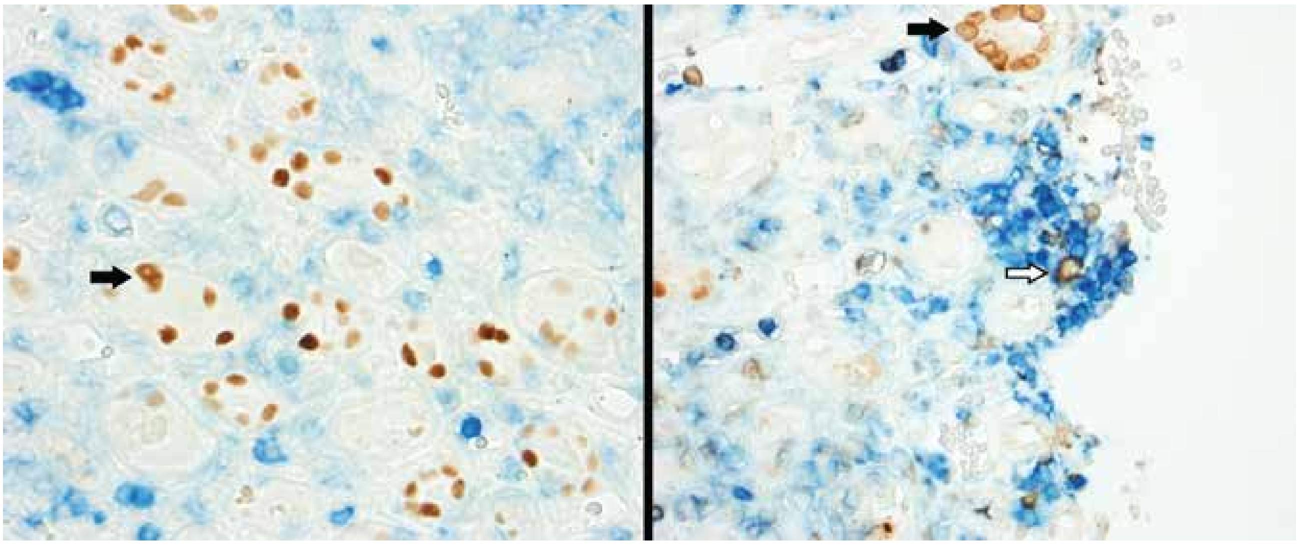

Supplementary Figure S12: IGV Visualization of BK Virus Strain, Variant Distribution, and Absence of Cross-Contamination

A. Integrated Genomics Viewer (IGV) visualization of BAM files for patient samples T68 and T105. The blue-colored reads are used for Unique Molecular Identifier counting. The coverage track (gray bars) represents the sequencing depth at each genomic position. BKV gene annotations are displayed. All observed variants appear in 100% of the reads, suggesting that each patient is infected with a single BKV strain.

B. Zoomed-in IGV view of the 3' end of the VP1 gene, showing patient-specific variants. T68 harbors four variants, while T105 has one variant. The absence of shared variants between the samples suggests no evidence of cross-contamination during sequencing or library preparation.

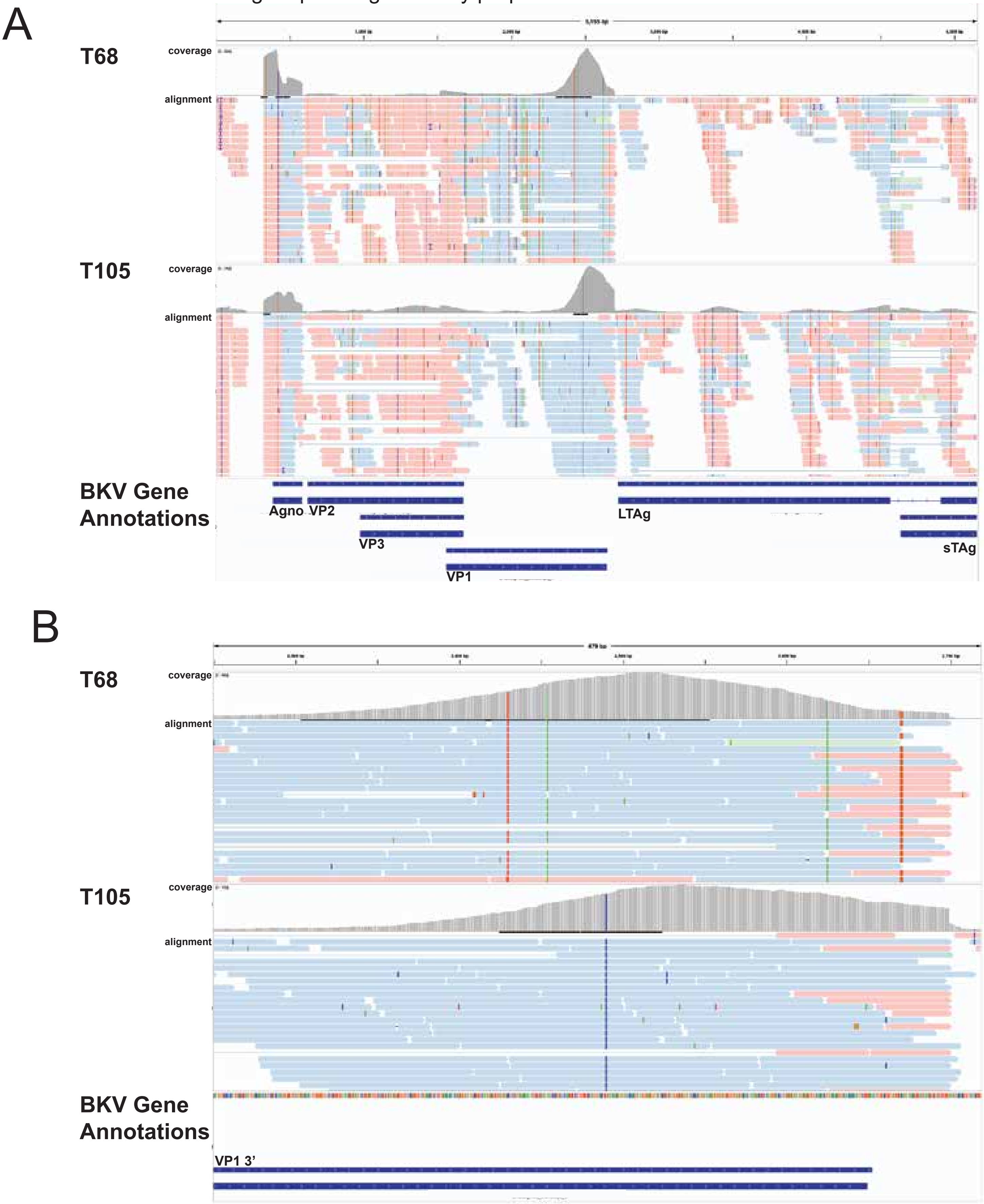

Supplementary Figure S13: BK viral particles are detectable in patients.
Top left: Electron dense arrays of replicated BK virus in the nucleus of a degenerating tubular epithelial cell. Top right: Detail of a viral array in a tubular epithelial cell showing heterogeneous electron dense rounded virions ranging in size from 40-54 nm. Bottom left: Nearby interstitium containing leukocytes infiltrating around collagen matrix. Bottom right and inset: Detail of a leukocyte showing rounded particles of similar shape, electron density, and size to BK within an intracellular compartment. The image may represent phagocytosis of viral particles by a macrophage. These particles are present in 3/9 likely macrophages, 0/11 likely lymphocytes, and 0/9 plasma cells from selected fields of 2 levels of 1 epoxy block. The image is 400x.

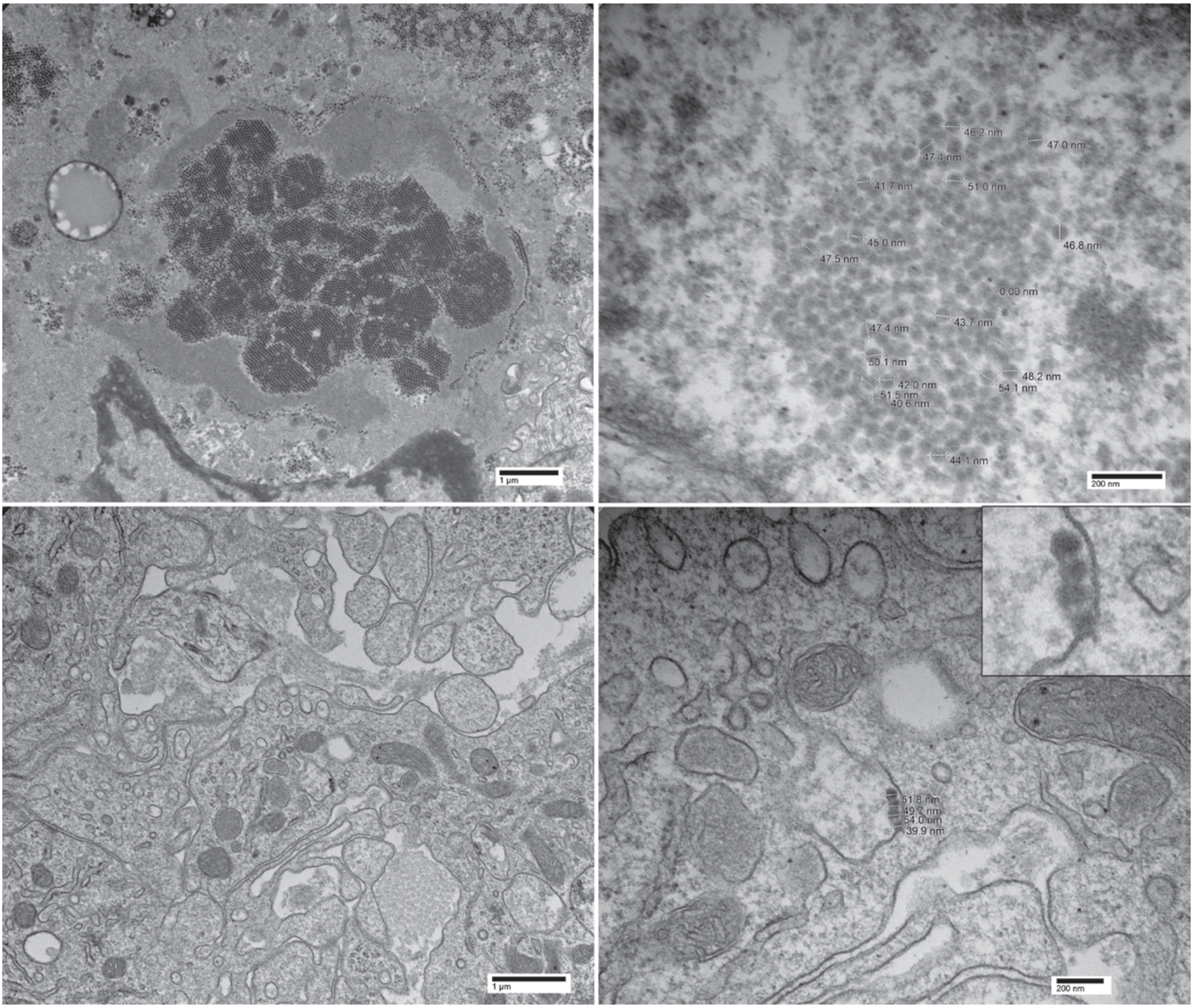

Supplement: Supplemental data [file jciinsight-11-198227-s011.pdf]
